# Selective vulnerability of tripartite synapses in amyotrophic lateral sclerosis

**DOI:** 10.1007/s00401-022-02412-9

**Published:** 2022-03-19

**Authors:** Matthew J. Broadhead, Calum Bonthron, Julia Waddington, William V. Smith, Maite F. Lopez, Sarah Burley, Jessica Valli, Fei Zhu, Noboru H. Komiyama, Colin Smith, Seth G. N. Grant, Gareth B. Miles

**Affiliations:** 1 School of Psychology and Neuroscience, University of St Andrews, St Andrews, UK; 2 Edinburgh Super-Resolution Imaging Consortium, Heriot Watt University, Edinburgh, UK; 3 Genes to Cognition Program, Centre for Clinical Brain Sciences, University of Edinburgh, Edinburgh, EH16 4SB UK; 4 Simons Initiative for the Developing Brain (SIDB), Centre for Discovery Brain Sciences, University of Edinburgh, Edinburgh, EH8 9XD UK; 5 Centre for Clinical Brain Sciences, University of Edinburgh, Edinburgh, UK; 6 Euan MacDonald Centre for MND Research, The University of Edinburgh, Edinburgh, UK

**Keywords:** ALS/MND, Synapse, Astrocyte, Neurodegeneration

## Abstract

Amyotrophic Lateral Sclerosis (ALS) is a fatal neurodegenerative disorder. Separate lines of evidence suggest that synapses and astrocytes play a role in the pathological mechanisms underlying ALS. Given that astrocytes make specialised contacts with some synapses, called tripartite synapses, we hypothesise that tripartite synapses could act as the fulcrum of disease in ALS. To test this hypothesis, we have performed an extensive microscopy-based investigation of synapses and tripartite synapses in the spinal cord of ALS model mice and post-mortem human tissue from ALS cases. We reveal widescale synaptic changes at the early symptomatic stages of the SOD1^G93a^ mouse model. Super-resolution microscopy reveals that large complex postsynaptic structures are lost in ALS mice. Most surprisingly, tripartite synapses are selectively lost, while non-tripartite synapses remain in equal number to healthy controls. Finally, we also observe a similar selective loss of tripartite synapses in human post-mortem ALS spinal cords. From these data we conclude that tripartite synaptopathy is a key hallmark of ALS.

**Supplementary Information:**

The online version contains supplementary material available at 10.1007/s00401-022-02412-9.

## Introduction

Amyotrophic Lateral Sclerosis (ALS) is a devastating form of motor neuron disease, characterised by loss of motor neurons (MNs) in the brain, brainstem and spinal cord, leading to progressive decline of motor control and fatal paralysis, typically within a few years of diagnosis [85]. The pathological mechanisms that precede MN cell death remain unclear. One line of evidence suggests that ALS, such as many neurodegenerative disorders, could be caused by a loss or a change in the synapses between neurons within the central nervous system (CNS) prior to cell loss [23, 31, 75, 78]. Pre-symptomatic and early symptomatic changes in cortical dendritic spines, neuromuscular junctions and the synaptic inputs to spinal cord MNs have all been reported [24, 25, 60, 63, 68, 72, 75]. Separate evidence indicates that astrocytes, the supportive glial cells of the CNS, may be responsible for non-cell autonomous degeneration of MNs [17, 26, 74, 81, 82, 86]. Astrocytes make specialised connections with chemical synapses, forming tripartite synapses, through which they help regulate synaptic structure and transmission [2, 59]. Despite evidence that glial cells may contribute to synaptic degeneration in neurodegenerative diseases [5, 39, 71], it is not known whether tripartite synapses are affected in ALS. We hypothesise that both synaptic and astrocytic dysfunction could be mechanistically linked, and that the tripartite synapse could act as a vulnerable fulcrum of disease in ALS.

To investigate how synapses and tripartite synapses are affected in ALS, we have performed a thorough, quantitative microscopy-based study of millions of excitatory synapses and markers of perisynaptic astrocytic processes (PAPs) in the spinal cord. This was conducted in mice with ALS-causing mutations and in post-mortem spinal cord tissue from human ALS patients. Using high-resolution and super-resolution microscopy, in combination with genetic and immunohistochemical labelling strategies for visualising synapses and astrocytes, our findings reveal that tripartite synapses are selectively vulnerable to degeneration in ALS. This finding suggests that synaptic loss may be mechanistically linked to astrocytic pathology in ALS, and the identification of this vulnerable synapse subtype could pave the way for targeted therapeutic strategies.

## Methods

### Animals and ethics

All procedures performed on animals were conducted in accordance with the UK Animals (Scientific Procedures) Act 1986 and were approved by the University of St Andrews Animal Welfare and Ethics Committee. The following mouse lines were kindly provided by Dr Richard Mead, University of Sheffield: B6SJL-TgN(SOD1-G93A)1Gur/J (SOD1^G93a^), B6SJL-Tg(SOD1)2Gur/J (WTSOD1) and FVB/NJ-Tg(C9ORF72)500Lpwr/J (C9orf72). SOD1^G93a^ and WTSOD1 were maintained on a congenic C57bl/6 background strain, while C9orf72 mice were maintained on an FVB background mouse strain. The ALS mouse lines were crossed with PSD95-eGFP^+/+^ mice, originally obtained from Prof. Seth Grant (University of Edinburgh), to produce transgenic (Tg) ALS and control offspring expressing PSD95-eGFP^+/-^. Unlike the other mouse lines, C9orf72 mice were produced on an FVB background. The progeny of C9orf72 × PSD95-eGFP mice were a first generation FVB/C57bl/6 cross, displaying a light brown fur coat and black eyes (indicating they did not possess the recessive retinal degeneration 1 allele of Pde6brd1 which causes blindness in FVB mice).

Disease phenotype was monitored by routine weighing and behavioural scoring from the age of 30 days (P30), with more frequent checks performed once the mice were over P60 or when mice were showing disease phenotypes. Mice were scored 0–4 in severity; 4 showing no phenotype, 3 showing hind-limb splay and tremors upon tail raising, 2 showing gait abnormalities, 1 showing hind limb dragging, 0 showing inability to self-right after 30 s when placed on its back. Mice were euthanised through either cervical dislocation or perfusion if displaying a score of 2 or lower.

Overall, SOD1^G93a^ mice presented hind limb tremor and reduced hind-limb splay by 76 days. We observed no difference in the age of symptom onset between SOD1^G93a^ mice and the cross-bred PSD95-eGFP mice, nor have we observed any difference in the age of symptom onset within our colonies over time. Our data is remarkably consistent with those of the source colonies [53] and suggests that little-to-no reduction in transgene copy number had occurred within our breeding colonies or experimental cohorts. No such overt phenotypes were observed in either the WTSOD1 or C9orf72 mouse colonies.

### Mouse tissue collection

Mice were anaesthetised with pentobarbitol (30 mg/kg dose; Dolethal), and the chest cavity opened to reveal the heart. The right atrium was severed and 10 ml ice cold 1 × phosphate buffered saline (PBS) was perfused through the left ventricle, followed by 10 ml 4% paraformaldehyde (PFA; Alfa Aesar). The lumbar spinal cord was then dissected and incubated for a further 3–4 h in 4% PFA before being incubated in sucrose 30% *w*/*v* for 24–72 h at 4 °C until sunk. Tissue was then cryo-embedded in OCT compound and stored at − 80 °C. Cryosections were obtained using a Leica CM1860 cryostat at 20 μm thickness and adhered to Superfrost Gold Plus glass slides (VWR).

### Mouse tissue processing and immunohistochemistry

Mouse tissue processing for visualising synapses and tripartite synapses is detailed in Broadhead et al. [6]. For visualisation of PSD95-eGFP only, slides were thawed at room temperature, briefly washed in PBS then deionised (DI) water before being dried and mounted in lab-made Mowiol or Prolong Glass anti-fade for super-resolution microscopy with a 1.5 thickness coverslip. For immunohistochemistry, sections were first heated at 37 °C for 30 min to aid the adherence of the tissue to the glass slides and reduce tissue loss during subsequent wash steps. Slides were washed three times in PBS before being blocked and permeabilised in PBS containing 3% Bovine Serum Albumin (BSA) and 0.2% Triton X100 for 2 h at room temperature. Primary antibody incubation was performed in PBS containing 1.5% BSA, 0.1% Triton X100 for 2 nights at 4 °C. Once incubated, slides are washed five times in PBS over the course of 1–2 h. Secondary antibody incubation was performed in 0.1% Triton X100 for 2 h at room temperature followed by a further five washes in PBS over the course of 1–2 h. Dye labelling of astrocyte-specific primary antibodies (GFAP, EAAT2 and p-Ezrin) was performed with 10 µg of each antibody using the Lightning-Link conjugation kits (Abcam) to address whether some proteins colocalised in astrocytes when the primary antibodies were raised in the same species (rabbit). See Table 1 for details on antibodies for mouse tissue experiments.

**Table 1:** Antibodies for mouse spinal cord tissue labelling

Primary antibody target	Species	Company	Code	Dilution	Secondary antibody/conjugation
VGLUT2	Mouse	Abcam	Ab79157	1:500	Anti-Ms Alexa Fluor 555
p-Ezrin	Rabbit	ProteinTech	26056-1-AP	1:500	Anti-Rb Alexa Fluor 647 / Dye-Conjugated Alexa Fluor 647
EAAT2	Rabbit	Bioss	BS1751R	1:500	Anti-Rb Alexa Fluor 647 / Dye-Conjugated Alexa Fluor 594
zDHHC2	Rabbit	Biorbyt	Orb385443	1:100	Anti-Rb Alexa Fluor 647
Glutamine Synthetase	Mouse	ProteinTech	66323-1-Ig	1:500	Anti-Ms FITC
ChAT	Chicken	Abcam	Ab34419	1:250	Anti-Ch Alexa Fluor 647
GFAP	Rabbit	DAKO	Z0334	1:1000	Dye-conjugated Alexa Fluor 594

### Human post-mortem tissue and immunohistochemistry

Human cervical spinal cord samples were obtained from the Edinburgh Brain and Tissue Bank, by Prof. Colin Smith. Ethical approval for this work was provided by the East of Scotland Research Ethics Service REC 1 (16/ES/0084). Samples of the cervical spinal cord were obtained from patients who died from ALS displaying mutations in the C9orf72 gene or the SOD1 gene. Age matched patients who died from natural causes were used as controls. Mean average patient ages were 57.2 years (controls), 57.6 years (C9orf72) and 54 years (SOD1). The immunohistochemistry protocol used was adapted from Curran et al. [15]. Sections were fixed, paraffin embedded and sectioned at 4 μm thickness and mounted to glass slides. For immunohistochemistry, samples were first de-paraffinised with sequential 3 min washes in Xylene, 100% ethanol, 95% ethanol, 70% ethanol and 50% ethanol. Antigen retrieval was performed by incubating samples in Citric Acid (pH 6.0) for 20 min in a steamer, providing a consistent heat of 95 °C. Following antigen retrieval, sections were washed three times in PBS and then incubated in 1 × Tris buffered Saline (TBS) with 5% BSA, 0.2% Triton X100 for 2 h at room temperature. Samples were then incubated in primary antibody solution consisting of TBS with 3% BSA, 0.2% Triton X100 for two nights at 4 °C before being washed five times in TBS containing 0.2% Triton X100 over the course of 1 h. Secondary antibody incubation was performed in TBS with 0.2% Triton X100 solution for 3 h at room temperature followed by 5–6 washes in PBS with 0.2% Triton X100 over the course of 2 h. Finally, sections were washed in DI water, dried and mounted in Mowiol. See Table 2 for antibodies used in human tissue experiments.

**Table 2:** Antibodies for human spinal cord tissue labelling

Primary antibody target	Species	Company	Code	Dilution	Secondary antibody/conjugation
PSD95	Guinea Pig	Synaptic Systems	124-014	1:500	Gt Anti-Gp Alexa Fluor 647
p-Ezrin	Rabbit	ProteinTech	26056-1-AP	1:250	Gt Anti-Rb Cy3
VGLUT2	Mouse	Abcam	Ab79157	1:500	Dk Anti-Ms Alexa Fluor 555

### RNA FISH

Cy5-labelled DNA probes were used against the G4C2 RNA repeat expansion. All solutions were made up in DEPC-treated water or PBS. Fixed spinal cord tissue was permeabilised with 0.1% Triton X100 for 1 h. Tissue was treated with 2 × saline sodium citrate (SSC) and 10% formamide in PBS for 5 min. Slides were then incubated with hybridization solution containing probes in 10% formamide overnight at 37 °C. After hybridization slides were washed once in 2 × SSC containing 10% formamide, for 30 min at 37 °C, then washed in 2 × SSC for 5 min at room temperature. DAPI was used at 1:15,000 in PBS. Images were captured on a Zeiss Airyscan LSM800 at 63x.

### High-resolution microscopy

High-resolution confocal-like microscopy was performed using a Zeiss Axio Imager M2 Microscope equipped with an Apotome 2.0, which enables an XY resolution of 320 nm [6]. Illumination was provided by a HXP120 lamp, and images acquired using a digital MRm camera. Exposure times and illumination intensity were kept consistent for analysis within batches of data sets. To ‘map’ entire hemi-sections of spinal cords to study anatomical diversity between laminae, single optical sections were captured and tiled across half a transverse section of the spinal cord. Maps were captured using a single *Z*-stack plane approximately 3–5 µm depth into the tissue that was kept at the same depth throughout the entire map. Images were stitched together to create whole montage images that were subsequently analysed.

### Super-resolution microscopy

Confocal and gated-stimulated emission depletion (g-STED) microscopy was performed using a Leica SP8 SMD g-STED microscope available at the Edinburgh Super-Resolution Imaging Consortium hosted by Heriot Watt University. Excitation was provided by a CW super-continuum white light laser source at 488 nm to excite eGFP with depletion provided by a 594 nm and 775 nm laser. Images were acquired with a 100 × 1.4NA STED objective lens with an optical zoom set to provide optimal *xy*-resolution.

### Image analysis

Image analysis was performed in Fiji is just ImageJ (FIJI) [66]. High-resolution image analysis was performed as described previously [6]. A standard mouse spinal cord anatomical atlas was used to guide the delineation of Rexed’s laminae [77]. For clarity, our delineation of lamina VIII represents a heterogenous population of ventral neurons including medial MN pools, while laminae IX represents lateral MN pools. For mapping data sets, whole images were processed using a background subtraction and gaussian smoothing. For the PSD95-eGFP-only data sets of excitatory synapse mapping, automated Moments-based thresholding was applied. For data sets involving detection of immunolabelled markers of synaptic or astrocytic structures, manual thresholding was typically required, and the researcher was blinded to the genotype of the mouse to avoid unconscious bias. Minimum and maximum size thresholds were set to minimise false positive detections of small dim structures of a few pixels in size and large clusters that were likely to be lipofuscin aggregates. Colocalisation between structures in high-resolution data sets was determined from binarized images with the criteria of showing overlap by at least 1 pixel.

Nanostructural analysis of PSD95 organisation from g-STED imaging was analysed as described previously [6, 7]. Briefly, confocal and g-STED images are processed with background subtraction and gaussian smoothing. Structures resolved in g-STED were deemed to be PSD95 nanoclusters (NCs), while larger diffraction-limited structures resolved with confocal microscopy were deemed to be whole PSDs. Structural parameters such as size, shape and intensity were obtained for PSDs and NCs, as well as how many NCs resided within a given PSD.

### Statistical analysis

**Fig. 1:**
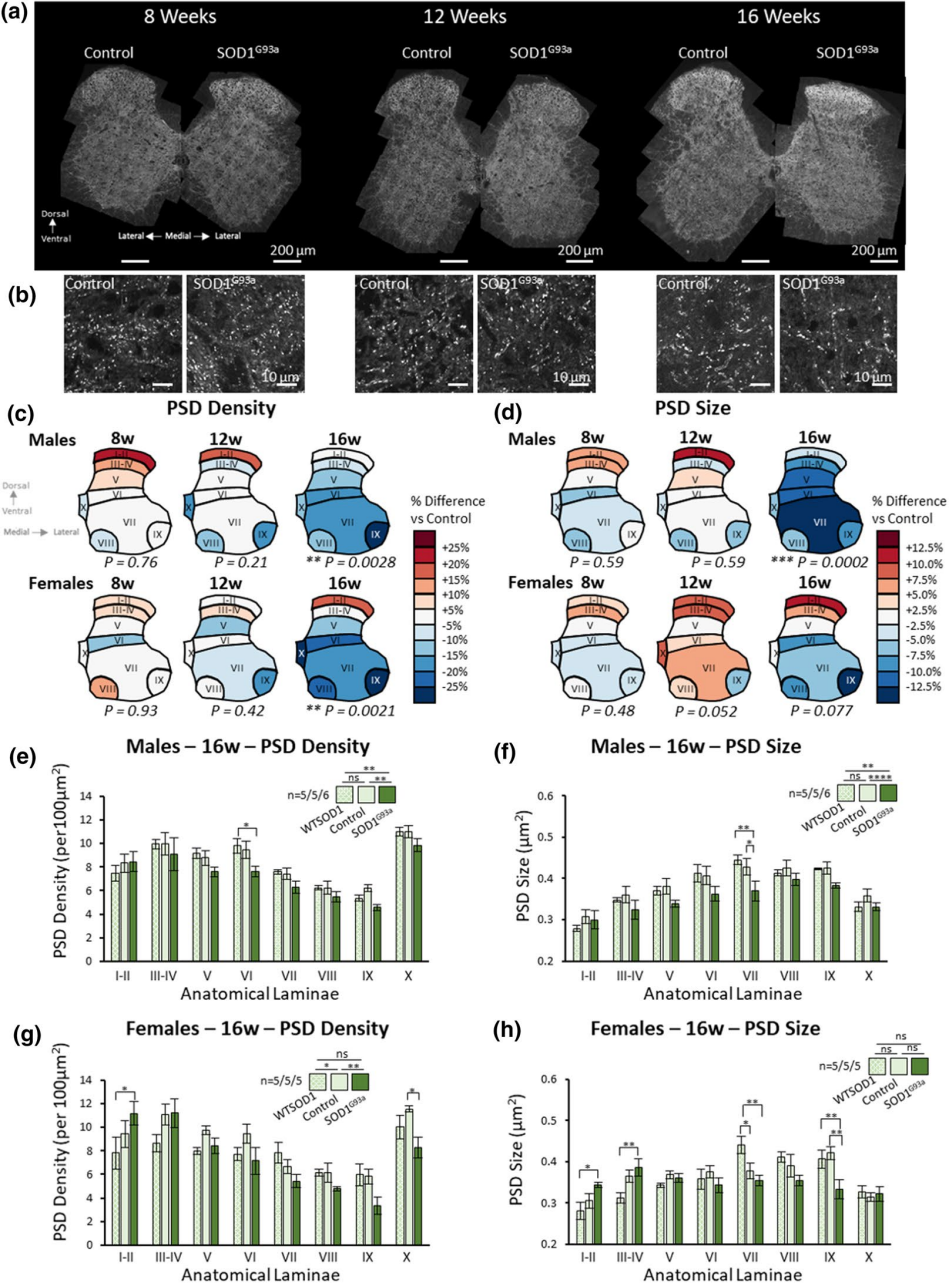
Mapping excitatory synapses in the spinal cord revealed early symptomatic stage synaptic changes in SOD1^G93a^ mice. **a** High-resolution mapping of PSD95-eGFP was performed to analyse synaptic changes in SOD1^G93a^ mice at different time points of the disease. **b** Example images of postsynaptic puncta in control and SOD1^G93a^ mice at different time points of disease. **c** Heat maps of PSD density in male and female SOD1^G93a^ mice compared to their respective controls at different ages. **d** Heat maps of PSD size in male and female SOD1^G93a^ mice compared to their respective controls at different ages. **e** Chart plotting PSD density in all 8 laminae of 16-week-old controls, WTSOD1 controls and SOD1^G93a^ males. Sample sizes denoted as *n* = number of WTSOD1’s/number of controls/number of SOD1^G93a^’s. **f** Chart plotting PSD size in all 8 laminae of 16-week-old controls, WTSOD1 controls and SOD1^G93a^ males. **g** Chart plotting PSD density in all 8 laminae of 16-week-old controls, WTSOD1 controls and SOD1^G93a^ females. **h** Chart plotting PSD size in all 8 laminae of 16-week-old controls, WTSOD1 controls and SOD1^G93a^ females

**Fig. 2:**
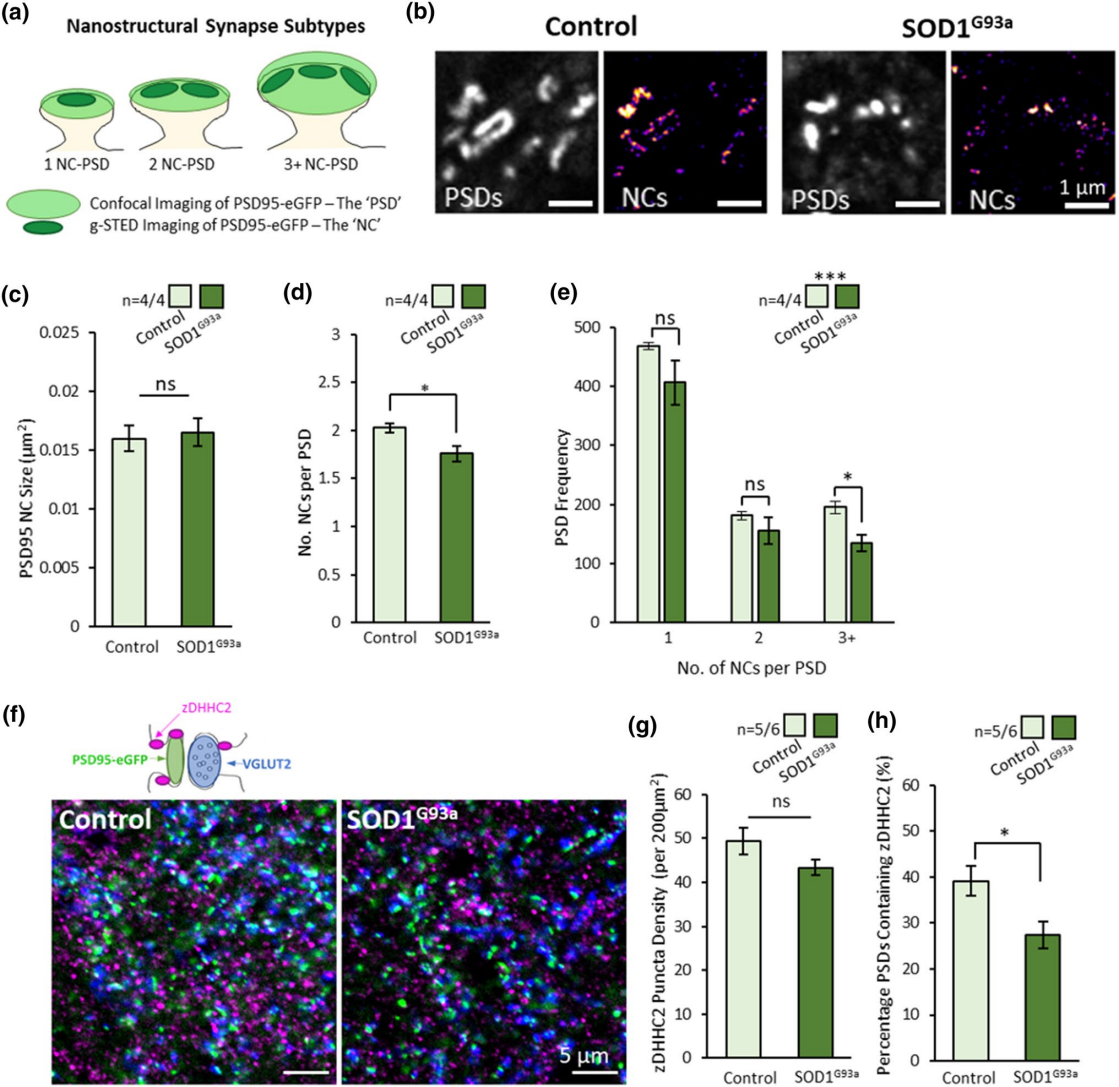
Nanoscopic Changes in Substructural Organisation of PSD95 in ALS Synapses. **a** Diagram of synapse subtypes, defined by the number of PSD95 nanoclusters (NCs) per PSD. PSDs can be visualised using confocal microscopy while NCs are resolved from correlative g-STED images. **b** Spinal cord tissue from control and SOD1^G93a^ mice were imaged using confocal and g-STED microscopy to visualise PSDs and NCs, respectively. **c** Graph showing the size of the PSD95 NCs was not different between controls and SOD1^G93a^ mice. **d** Graph showing the average number of NCs per PSD was significantly reduced in SOD1^G93a^ mice. **e** Graph showing the number of large 3 + NC-PSDs are specifically reduced in SOD1^G93a^ mice compared to controls. **f** High-resolution imaging of synapses (PSD95 and VGLUT2) and the palmitoylation enzyme, zDHHC2, was performed to investigate whether changes in zDHHC2 expression might explain the reduced number of PSD95 NCs at synapses. **g** Graph showing no difference in zDHHC2 puncta density between control and SOD1^G93a^ mice. **h** Graph showing reduced number of PSDs expressing zDHHC2 in SOD1^G93a^ mice

**Fig. 3:**
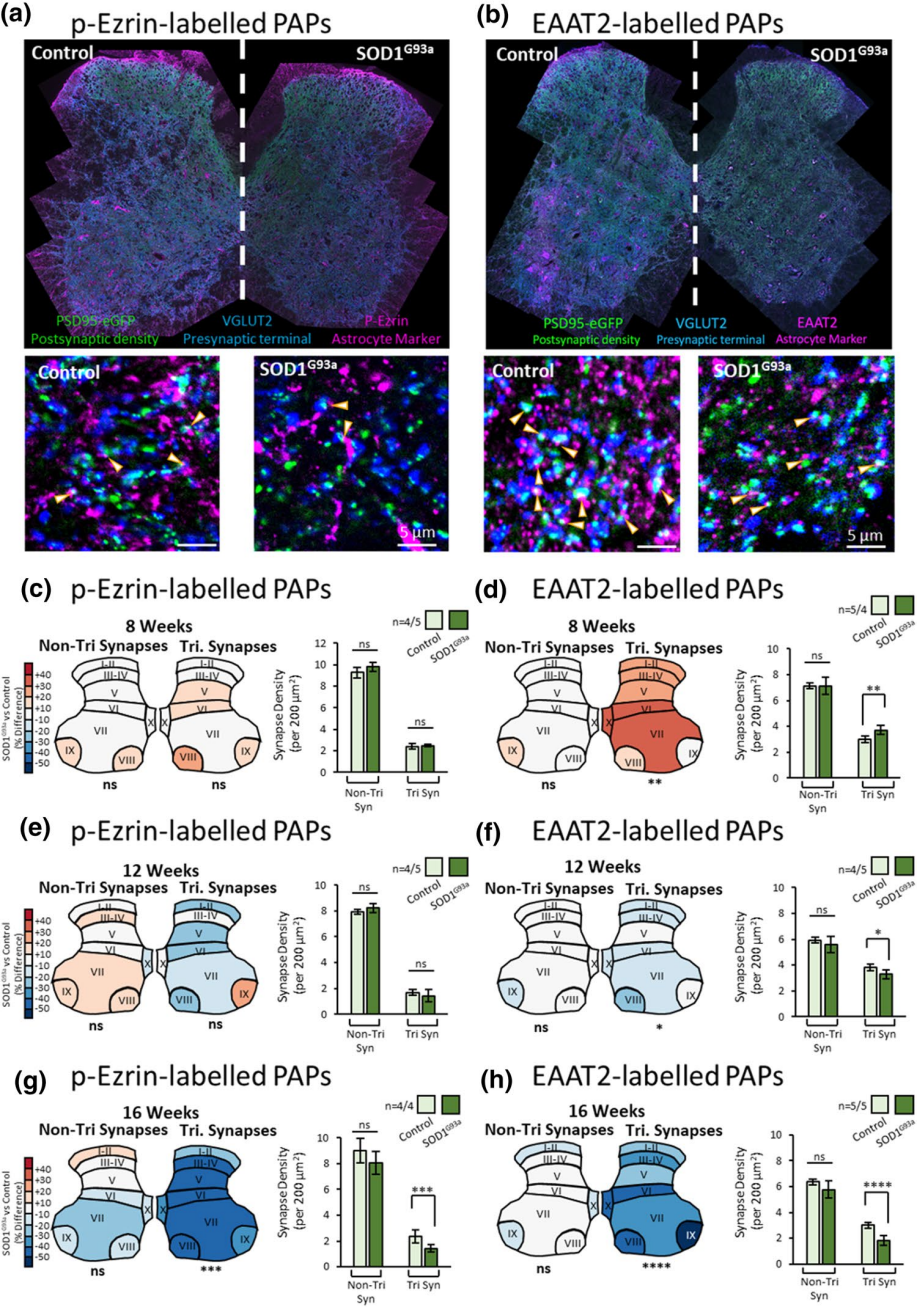
Mapping Tripartite Synapses in the SOD1^G93a^ Mouse Spinal Cord. **a** Example of high-resolution spinal cord mapping of tripartite synapses from 16-week-old male control and SOD1^G93a^ mice using PSD95-eGFP, VGLUT2 and p-Ezrin to label the PSDs, presynaptic terminals and PAPs, respectively. Cropped images display individual synaptic and tripartite synaptic structures (denoted by arrows). **b** Example of high-resolution spinal cord mapping of tripartite synapses from 16-week-old male control and SOD1^G93a^ mice using PSD95-eGFP, VGLUT2 and EAAT2 to label the PSDs, presynaptic terminals and PAPs, respectively. Cropped images display individual synaptic and tripartite synaptic structures (denoted by arrows). **c** Colour coded heat maps denote the difference in synapse or tripartite synapse density observed in SOD1^G93a^ mice as a percentage of the respective controls. Bar charts display the mean synapse density from mice, averaged across all anatomical subregions for simplicity. From p-Ezrin labelling, no changes in non-tripartite or tripartite synapses were observed in 8 week SOD1^G93a^ mice. **d** From EAAT2 labelling, no changes in non-tripartite synapses were observed, but a significant increase in tripartite synapses was observed in 8 week SOD1^G93a^ mice compared to control. **e** From p-Ezrin labelling, no changes in non-tripartite or tripartite synapses were observed in 12 week SOD1^G93a^ mice. **f** From EAAT2 labelling, no changes in non-tripartite synapses were observed, but a significant decrease in tripartite synapses was observed in 12 week SOD1^G93a^ mice compared to control**. g** From p-Ezrin labelling, no changes in non-tripartite synapses were observed, but a significant decrease in tripartite synapses was observed in 16 week SOD1^G93a^ mice compared to control. **h** From EAAT2 labelling, no changes in non-tripartite synapses were observed, but a significant decrease in tripartite synapses was observed in 16 week SOD1^G93a^ mice compared to control

**Fig. 4:**
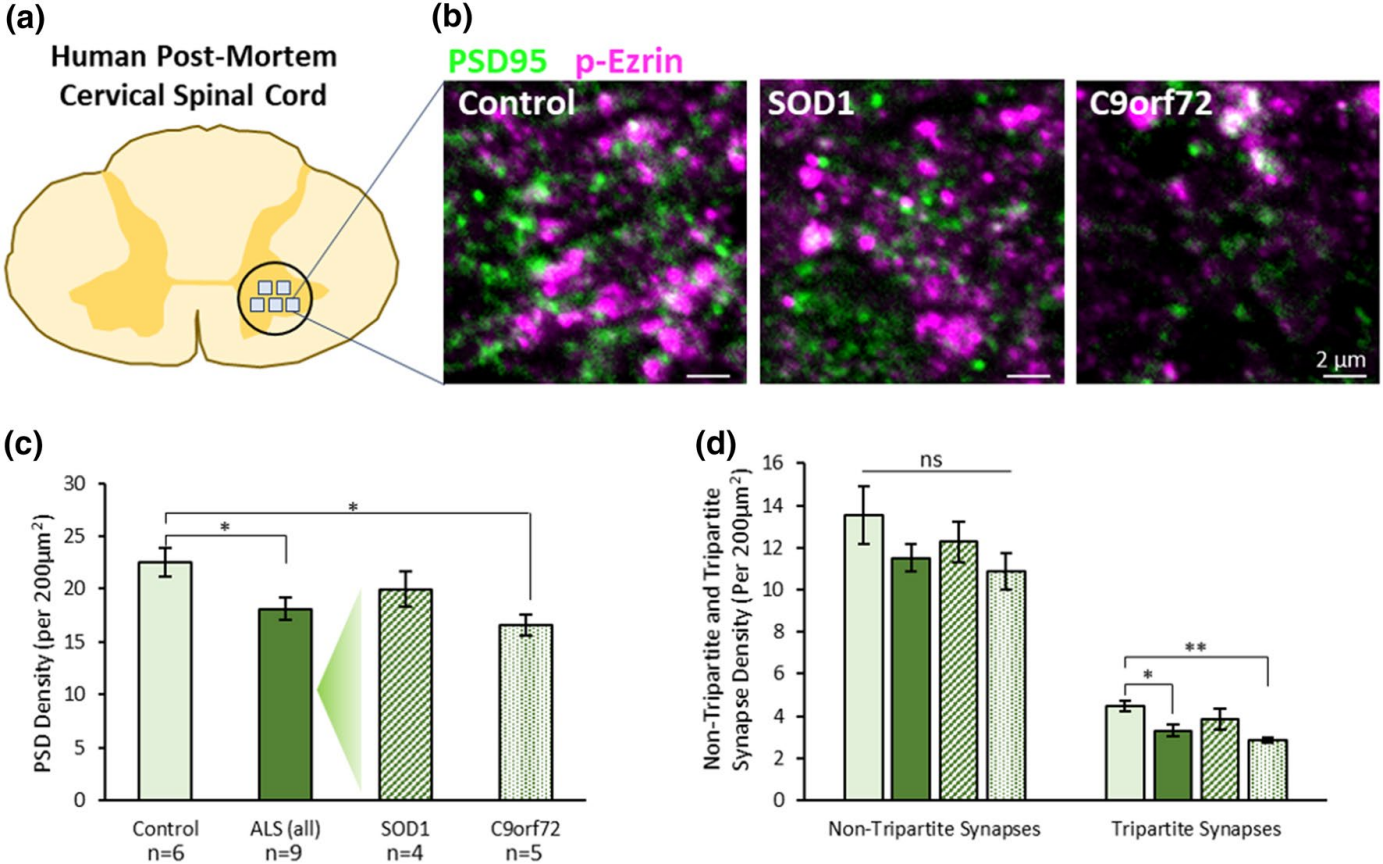
Tripartite Synapses are selectively lost in the cervical spinal cord of human ALS cases. **a** Diagram of human cervical spinal cord and location of image sampling. **b** Example images of synapses (PSD95—green) and astrocytic processes (p-Ezrin—magenta) in human spinal cord tissue. **c** Graph showing the total PSD density (PSD95 puncta) in controls, all ALS cases, and then SOD1 and C9orf72 cases separated. **d** Graph showing synapse density in patient groups when synapses are separated based on whether they are non-tripartite synapses or tripartite synapses, as determined by the association of PSD95 with p-Ezrin

Data analysis and graph preparation was performed in Microsoft Excel. Statistical analyses were performed using either SPSS (IBM) or Prism (Graphpad). Data were assessed for normality using the Shapiro Wilks test. Multi-factorial (Two-Way) ANOVA were used for mouse spinal cord synaptic and tripartite synaptic mapping (data presented in Figs. 1 and 3, SI Figs. 3, 6 and 7), where the factors were genotype and anatomical subregion. Sulak’s post-hoc comparison test was performed following Two-Way ANOVA’s, where more than two genotypes (e.g., controls, SOD1^G93a^ and WTSOD1 mice) were compared, while Tukey’s post-hoc comparison was performed following Two-Way ANOVA’s, where only two groups were compared (e.g., controls and SOD1^G93a^ mice). Two Sample *T*-tests were performed for PSD95 nanoscale analyses (presented in Fig. 2c–e), zDHHC2 expression (Fig. 2g, h), ChAT MN staining quantification (SI. Fig. 2b, c) and human ALS synapse analysis, when both SOD1 and C9orf72 patient cases were merged into one ‘ALS’ group and compared with controls (Fig. 4c,d). Pearson’s Chi-squared test was performed to test for differences in the frequency of synapse subtypes in controls and SOD1^G93a^ mice, in combination with Two-Sample T-Tests to compare the frequency of each specific synapse subtype between the two genotypes (Fig. 2e). Pearson’s Correlation Analysis was performed to assess regional differences in tripartite synapse loss and the percentage of synapses contacted by astrocytes (SI. Figure 7). One-Way ANOVAs with post-hoc Tukey’s comparison tests were used to assess differences in synapses and tripartite synapses between Control, SOD1 and C9orf72 human patient spinal cords (Fig. 4c,d).

## Results

### Widescale mapping of the mouse spinal cord reveals synaptic changes in ALS

To visualise and quantify synaptic changes in ALS mice using a standardised and robust approach, we cross-bred PSD95-eGFP^+/+^ mice [7, 87] with SOD1^G93a^ mice [35], WTSOD1 mice [35] and C9orf72 mice [48]. The use of PSD95-eGFP mice ensures that all mice expressed fluorescently tagged postsynaptic density (PSD) scaffolding molecule, PSD95, at virtually all excitatory synapses throughout the nervous system [87]. As sex-dependent differences in phenotypes have been observed in both the SOD1^G93a^ and C9orf72 mice [10, 36, 48], males and females were analysed separately.

From here onwards, the terms SOD1^G93a^, WTSOD1 and C9orf72 refer to mice produced from breeding with homozygous PSD95-eGFP knock-in mice to facilitate synapse visualisation in heterozygous PSD95-eGFP progeny. The term ‘Control’ is used to refer to heterozygous PSD95-eGFP progeny not expressing the SOD1^G93a^ or C9orf72 transgene.

Consistent with previous reports [53], SOD1^G93a^ mice presented with hind-limb tremor and hind-limb splay by approximately 76 days of age, with males displaying a slightly earlier onset compared to females, though this was not statistically significant (73.5 days for *n* = 4 male SOD1^G93a^ mice, 77.1 days for *n* = 9 female SOD1^G93a^ mice; *t*(11) = 1.3, *p* = 0.21).

At the point of termination, none of the 8-week SOD1^G93a^ mice displayed hind limb tremor (5 males, 5 females); all the 12-week male (5/5) and some of the 12-week female (3/5) SOD1^G93a^ mice displayed hind limb tremor, and all the 16-week mice SOD1^G93a^ (6 males, 5 females) displayed hind limb tremor. 16-week old SOD1^G93a^ mice were also lower in weight compared to controls (males: *t*(9) = 5.2, *p* = 0.0006; females: (*t*(8) = 5.3, *p* = 0.0007). No signs of motor dysfunction or weight loss were observed in WTSOD1 mice up to 16 weeks. Similarly, no signs of acute motor dysfunction or weight loss were seen in C9orf72 mice up to 22 weeks. C9orf72 mice did display G4C2 RNA foci, a key molecular hallmark of disease, in the nuclei of spinal cord cells (SI. Fig. 1a, b).

Tiled, high-resolution maps of PSD95-eGFP synapses were obtained across hemisects of spinal cords which we delineated into spinal laminae I–II, III–IV, V, VI, VII, VIII, IX and X. Each map of a mouse hemi-spinal cord yielded data on approximately 40,000–50,000 PSDs. In total, spinal cord synapse maps were obtained from 122 mice, from which over 5 million PSDs were quantified. In this study, our analysis focuses on PSD density (the number per unit area) and PSD size (area). Overall, inter-regional mapping of PSD95 revealed anatomical diversity in synapse number and size comparable to our previous observations [6], with little difference associated with age or sex.

In both male and female SOD1^G93a^ and control mice, synaptic maps were generated at 8 weeks (pre-symptomatic), 12 weeks (early symptomatic) and 16 weeks of age (early symptomatic with significant weight loss) (Fig. 1a, b). At 8 and 12 weeks of age there was no difference between SOD1^G93a^ and controls in PSD density (Fig. 1c) or PSD size (Fig. 1d). At 16 weeks of age, however, SOD1^G93a^ males showed significantly reduced PSD density (*F*(1,72) = 9.60, *p* = 0.0028) and PSD size (*F*(1,72) = 15.77, *p* = 0.0002) compared to controls. These effects were also significant when SOD1^G93a^ males were compared with both controls and WTSOD1 controls (Fig. 1e, PSD density: *F*(2,104) = 7.49, *p* = 0.0009; Fig. 1f, PSD size: *F*(2,104) = 11.02, *p* < 0.0001). These synaptic changes were widespread across the spinal cord, with the exception of the dorsal laminae I–II, while post-hoc Tukey’s testing revealed that PSD density and size were most significantly affected in lamina VI and VII, respectively.

The SOD1^G93a^ 16-week old females displayed reduced PSD density when compared to control females (*F*(1, 64) = 10.26, *p* = 0.0021), but no difference in PSD size (*F*(1, 64) = 3.23, *p* = 0.077). Furthermore, these effects were not significant in comparison to the female 16-week-old WTSOD1 mice (Fig. 1g, h). Interestingly, it was noted that unlike the SOD1^G93a^ males, the SOD1^G93a^ females displayed more complex laminae–genotype-specific synaptic changes in both PSD density (*F*(14, 96) = 2.34, *p* = 0.008) and size (*F*(14, 96) = 3.68, *p* < 0.0001). The dorsal laminae I-II of SOD1^G93a^ females displaying increased PSD density and size compared to WTSOD1, while ventral laminae were more likely to show reduced PSD density and size compared to WTSOD1 and controls.

To address whether synaptic loss was associated with MN cell death, we performed immunolabelling using a choline acetyltransferase (ChAT) specific antibody. We found no significant loss of MNs in 16-week old SOD1^G93a^ male spinal cords (SI. Fig. 2a–d; *t*(6) = 0.99, *p* = 0.36). While other studies indicate MN cell loss can occur from approximately 16 weeks onwards [19], our data suggest that synaptic changes occur prior to overt MN cell death.

Next, we examined the spinal cords of mice carrying a mutation in the C9orf72 gene, one of the most common genes associated with familial and sporadic ALS (SI. Fig. 3a, b). Synaptic maps from 12-week and 22-week-old C9orf72 mutant mice of both sexes were obtained to capture reported pre-symptomatic and early-symptomatic stages of the disease phenotype in this model [48, 55, 58]. Mapping PSD95-eGFP synapses revealed no difference in PSD density or size in C9orf72 mice compared to controls at either 12 or 22 weeks of age in both males and females (SI. Fig. 3c–j). The lack of synaptic changes in C9orf72 mice may reflect the fact that no motor phenotypes were observed in any of our C9orf72 mice even by 22 weeks. Our observations are consistent with reports from other groups that the penetrance of the phenotype in this mouse model can be very low in some colonies [55, 58].

In summary, our extensive synaptic mapping reveals a loss of PSDs at excitatory synapses in both male and female SOD1^G93a^ mice during early symptomatic stages. Our data also suggest that male SOD1^G93a^ mice display structural changes in synapses, an effect not as clearly observed in female SOD1^G93a^ mice, perhaps due to the delayed onset of disease in females. While we observed no synaptic changes in C9of72 mutant mice, this mirrors the fact that no ALS-related phenotypes, such as acute motor decline or weight loss, were observed in these mice. Given that our C9orf72 mice fail to robustly recapitulate an ALS disease phenotype, they were not included in any further analyses.

### Super-resolution microscopy reveals loss of multi-nanocluster synapses in the ALS mouse spinal cord

Our high-resolution microscopy data revealed both a reduced number and reduced average size of PSDs in 16-week SOD1^G93a^ males. While confocal-level resolution is sufficient to measure synapse number and overall PSD size, the nanoscale organisation of synapses is best resolved using super-resolution microscopy. PSD95 forms nanoclusters (NCs) of approximately 100–150 nm diameter within the PSD [6, 7, 49, 56]. PSD size, which correlates with synaptic strength, is determined by the number of NCs per PSD (Fig. 2a). We performed g-STED microscopy of PSD95-eGFP expression in 16-week-old male SOD1^G93a^ and control mice to quantify synaptic nanostructure (Fig. 2b; *n* = 4 mice per group). PSD95 NC size did not differ between genotypes (diameter ~ 140 nm; Fig. 2c; *t*(6) = 0.31, *p* = 0.77), and was consistent with previous measurements of PSD95 NC size using g-STED microscopy in both the brain and spinal cord [6, 7, 30]. Instead, SOD1^G93a^ mice showed a reduced average number of NCs per PSD (~ 1.75 NCs per PSD) compared with controls (~ 2 NCs per PSD) (Fig. 2d; *t*(6) = 2.8, *p* = 0.03). This appears to be the result of a significant loss of large multi-NC-PSDs in SOD1^G93a^ mice, while smaller 1 or 2 NC-PSDs were observed with similar frequency (Fig. 2e; *χ*(2) = 14.7, *p* = 0.0006). In controls, 23% of synapses were classed as containing three or more NCs, while 17% of SOD1^G93a^ synapses contained three or more NCs.

The formation of PSD95 NCs at the synapse is dependent on palmitoylation of PSD95 at its N-terminal cysteine residue by the palmitoylating enzyme, zDHHC2. Having revealed a reduced number of PSD95 clusters per synapse in ALS mice, we assessed whether a change in zDHHC2 expression could be responsible. We analysed excitatory synapses (PSDs using PSD95-eGFP and presynaptic terminals using anti-VGLUT2 immunolabelling) for their expression of zDHHC2 by acquiring high-resolution images from the ventral horn of spinal cords from 16-week male control and SOD1^G93a^ mice (Fig. 2f). zDHHC2 displayed a punctate expression showing close association with both PSD95-eGFP and VGLUT2 puncta. While the overall density of zDHHC2 puncta was not significantly different between genotypes (Fig. 2g; *t*(9) = 2.1, *p* = 0.067), there was a reduced number of PSDs colocalising with z-DHHC2 in SOD1^G93a^ mice (Fig. 2h; *t*(9) = 2.6, *p* = 0.031). This finding suggests that reduced postsynaptic presence of the palmitoylation enzyme, zDHHC2, may be related to a reduced number of PSD95 NCs at the synapse and lead to incremental reductions in postsynaptic strength.

### Widescale mapping reveals tripartite synapse loss in the ALS mouse spinal cord

Tripartite synapses are chemical synapses contacted by perisynaptic astrocytic processes (PAPs). Previously we used antibody labelling against PAP proteins EAAT2 and p-Ezrin to identify tripartite synapses in the mouse lumbar spinal cord based on colocalization or close association of PAP proteins with postsynaptic PSD95 and presynaptic VGLUT2 [6]. Immunolabelling for VGLUT2, p-Ezrin and EAAT2 are further validated here (SI. Figs. 4a–f, 5a, b). p-Ezrin and EAAT2 puncta strongly colocalised with one another in the mouse spinal cord (SI. Fig. 5a). Both PAP markers are closely associated with astrocytic cell bodies, branches and fine processes, labelled with glutamine synthetase, but display little direct colocalization with astrocytic GFAP-positive primary arbours (SI. Fig. 5b). These observations further confirm that both astrocytic EAAT2 and p-Ezrin label PAPs, making them ideal markers, in combination with synaptic markers, for visualising tripartite synapses. We, therefore, performed extensive high-resolution mapping of tripartite synapses in SOD1^G93a^ and control spinal cords, labelling PSDs (PSD95-eGFP), presynaptic terminals (VGLUT2) and PAPs (EAAT2 or p-Ezrin).

At 8 weeks, male SOD1^G93a^ mice displayed an increased density of EAAT2 puncta (*F*(1, 56) = 11.19, *p* = 0.0015; SI. Fig. 6a) and p-Ezrin puncta (*F*(1, 56) = 4.672, *p* = 0.035; SI. Fig. 6b) compared to controls. No changes were observed in male 12-week-old mice (SI. Fig. 6c, d), but by 16 weeks, male SOD1^G93a^ mice displayed significantly reduced EAAT2 (*F*(1, 64) = 11.31, *p* = 0.0013; SI. Fig. 6e) and p-Ezrin (*F*(1, 48) = 8.828, *p* = 0.0046; SI. Fig. 6f) puncta density compared to controls. These data demonstrate age-dependent changes in PAP proteins, suggesting an early stage increase in p-Ezrin-positive PAPs and increased EAAT2 expression at PAPs, followed by a significant loss of both proteins by 16 weeks, following the onset of early motor deficits.

We next quantified the density of non-tripartite synapses and tripartite synapses in male control and SOD1^G93a^ spinal cords at 8, 12 and 16 weeks. Using p-Ezrin, we observed no difference in the density of non-tripartite synapses between controls and SOD1^G93a^ males at either 8 weeks (Fig. 3c, *F*(1, 56) = 2.24, *P* = 0.1404) 12 weeks (Fig. 3e; *F*(1,56) = 0.56, *p* = 0.456) or 16 weeks of age (Fig. 3g; *F*(1,48) = 2.4, *p* = 0.126). The density of tripartite synapses was also no different between control and SOD1^G93a^ males at 8 weeks (Fig. 3c; *F*(1,56) = 0.2998, *p* = 0.586) and 12 weeks (Fig. 3e; *F*(1,56) = 0.83, *p* = 0.365). However, there was a significant reduction in tripartite synapse density at 16 weeks in SOD1^G93a^ males (Fig. 3g; *F*(1,48) = 14.59, *p* = 0.0004). It was also confirmed that female 16-week old SOD1^G93a^ mice displayed reduced numbers of p-Ezrin-associated tripartite synapses compared to controls (SI. Fig. 7a; *F*(1,80) = 5.65, *p* = 0.020), while non-tripartite synapses were seemingly unaffected (SI. Fig. 7b; *F*(1,80) = 0.19, *p* = 0.66).

Similarly, using EAAT2 as the PAP marker, the density of non-tripartite synapses was no different between controls and SOD1^G93a^ males at either 8 weeks (Fig. 3d; *F*(1, 56) < 0.0001, *p* = 0.99), 12 weeks (Fig. 3f; *F* (1, 56) = 1.37, *p* = 0.247) or 16 weeks (Fig. 3h; *F*(1,64) = 2.40, *p* = 0.126). The density of EAAT2-associated tripartite synapses, however, was significantly increased in 8-week SOD1^G93a^ males (Fig. 3d, *F*(1, 56) = 7.21, *p* = 0.0095), then subtly but significantly reduced in 12-week SOD1^G93a^ males (Fig. 3f; *F* (1, 56) = 4.77, *p* = 0.033) and more significantly reduced in 16-week SOD1^G93a^ males (Fig. 3h; *F* (1, 64) = 23.58, *p* < 0.0001). Female 16-week old SOD1^G93a^ mice also displayed reduced numbers of EAAT2-associated tripartite synapses compared to controls (SI. Fig. 7c; *F*(1,88) = 8.42, *p* = 0.0047), while non-tripartite synapses were unaffected (SI. Fig. 7d; *F*(1,88) = 3.48, *p* = 0.066).

Next, we investigated whether there is a relationship between synaptic vulnerability and astrocytic coverage of synapses in different spinal cord laminae (SI. Fig. 8a–d). In the 16-week old male SOD1^G93a^ mice, there was a significant correlation between the percentage of synapses contacted by p-Ezrin and the loss of tripartite synapses in each region (*r* = 0.935, *p* = 0.0006; SI. Fig. 8a, b). For example, dorsal laminae I–II displayed the highest proportion of synapses contacted by p-Ezrin (28%) and the smallest degree of tripartite synapse loss compared to controls (− 29%). This compares to ventral laminae VII with the smallest proportion of synapses contact by p-Ezrin (17%) and the greatest degree of tripartite synapse loss compared to controls (− 48%). There was a similar correlation between the percentage of EAAT2-associated tripartite synapses and the loss of tripartite synapses, though this was not statistically significant (*r* = 0.345, *p* = 0.401; SI. Fig. 8c, d).

From these experiments, using two different PAP markers, our data consistently demonstrate that tripartite synapses are a selectively vulnerable synapse subtype in early-symptomatic stage ALS mice.

### Selective loss of tripartite synapses in the human ALS spinal cord

Finally, we addressed whether human cases of ALS also demonstrated synaptic and tripartite synaptic degeneration. Post-mortem cervical spinal cord sections were obtained from human cases, including 6 healthy controls (5 male, 1 female), and 9 ALS patients (all male) comprising 4 SOD1 cases and 5 C9orf72 cases (SI. Table 1). Synapses and tripartite synapses were visualised in the ventral horn using immunolabelling for PSD95 and p-Ezrin to identify PSDs and PAPs, respectively (Fig. 4a, b; SI. Fig. 9a–f).

When comparing control cases with all ALS cases (SOD1 and C9orf72), PSD density was significantly reduced in the ALS cervical spinal cord (Fig. 4c; *t*(13) = 2.6, *p* = 0.021). Interestingly, when control, SOD1 and C9orf72 cases were analysed separately, only C9orf72 cases displayed significantly reduced PSD density compared to controls (*F*(2,12) = 5.4, *p* = 0.0214; Control versus C9orf72: *p* = 0.0167). We observed no difference in PSD size between the three groups (*F*(2,12) = 0.16, *p* = 0.85).

Next, we asked how tripartite synapses and non-tripartite synapses are affected in ALS patients (Fig. 4d). Similar to results from the SOD1^G93a^ mouse model, the density of non-tripartite synapses was no different between control, SOD1 and C9orf72 patients (*F*(2,12) = 1.44, *p* = 0.275), while the density of tripartite synapses was significantly reduced in C9orf72 cases compared to controls (*F*(2,12) = 8.16, *p* = 0.0058, Control versus C9orf72: *p* = 0.0044).

Taken together these findings provide evidence that tripartite synapses are selectively vulnerable to degeneration in human ALS cases, as observed in the SOD1^G93a^ mouse model.

## Discussion

This study set out to investigate how synapses and tripartite synapses are affected in the ALS spinal cord. To this end, we performed large-scale quantitative mapping of synapses and tripartite synapses in ALS mouse models and human post-mortem tissue. Our data revealed that, of the millions of synapses in the spinal cord, tripartite synapses appear to be selectively vulnerable to degeneration in ALS. We, therefore, describe ALS as a tripartite synaptopathy.

The functional, structural and molecular diversity of synapses throughout the nervous system, i.e., synaptome diversity, endows the nervous system with the capability to support a wide repertoire of behaviours from motor control to learning and memory [32, 33]. Over 130 different nervous system disorders, including ALS, can arise from mutations in postsynaptic proteins [4], and lead to wide-scale reprogramming of the synaptome [87]. Different diseases of the nervous system may display a unique synaptome signature, as certain subtypes of synapses may be more vulnerable to changes in one condition compared to another—thus leading to the specific behavioural consequences and symptoms of a given condition. By first identifying the vulnerable synapse subtypes of a given disease, we can establish therapeutic roadmaps to selectively target these synapse subtypes to treat the symptoms of disease.

The PSD95-eGFP mouse model provides a powerful tool with which to investigate the effects of diseases on synapses as PSD95 is abundantly expressed at the PSDs of excitatory synapses and forms a core molecular framework for synaptic machinery [13, 20, 28, 42, 87]. The same mouse model can be used for nanoscopic analyses of synapse subtypes based on postsynaptic substructure using super-resolution microscopy [6, 7].

This study also sought to identify whether astrocytes play a role in the vulnerability of synapses in ALS. Labelling the astrocytic component of the tripartite synapse was performed here using immunolabelling for either EAAT2 or p-Ezrin. Various studies support the use of either EAAT2 or Ezrin/p-Ezrin as robust markers for the PAPs which contact synapses [16, 27, 37, 46]. By performing tripartite synapse mapping with both PAP markers in parallel, we have observed strikingly similar findings which give us considerable confidence in our conclusion that tripartite synapses are vulnerable in ALS. Reduced levels of EAAT2 in the ALS spinal cord have been well documented [41, 47, 65]. The consistent reduction in both EAAT2 and the actin filament binding protein, p-Ezrin, suggest not just a molecular change in astrocytic protein expression but that the PAPs themselves are being lost.

Using the SOD1^G93a^ mouse model, we observed synapse loss, structural changes in PSDs, changes in PAPs and a loss of tripartite synapses at early, symptomatic stages. While we observed significant synaptopathy in SOD1^G93a^ mouse spinal cords, we observed no such alterations in C9orf72 mutant mice. The C9orf72 (C9BAC-500) mice were originally reported to display molecular hallmarks of ALS pathology, and acute motor deficits in 35% of the females carrying the mutation [48]. Further investigation by other groups using this model have shown different degrees of phenotypic penetrance [55, 58]. In our hands, the C9orf72 mice (the pure-bred colony and the progeny of PSD95-eGFP cross-breeding) display no such acute motor phenotypes in either males or females, though we did observe RNA foci accumulating in cell nuclei—a key molecular hallmark of ALS pathology. Other key molecular hallmarks of ALS such as TAR-DNA Binding protein-43 (TDP-43) pathology have been observed in the brains of symptomatic end-stage C9orf72 mice, but were not observed in asymptomatic mice [48], and this remains untested in the spinal cord. We consider that either genetic or environmental differences in our mouse colonies may have led to significantly reduced penetrance of the disease phenotype in the C9orf72 mouse line [58]. Given that phenotypic penetrance can be low, and that acute ALS phenotypes can occur between 20 and 40 weeks of age, further data collection from larger numbers of older animals may have captured the potential synaptic phenotype of this model. On the other hand, another mouse model with a more reliable phenotype, such as TDP-43^A315T^ which has been shown previously to display both synaptic and astrocytic changes [44, 45], may be a more suitable alternative for further investigation of tripartite synaptopathy.

We employed large-scale anatomical mapping of synapses and tripartite synapses in the lumbar mouse spinal cord. It was anticipated that some anatomical laminae, for example laminae VII–IX, where MNs reside, may display more significant changes in synapses in ALS. Despite significant inter-regional diversity in PSD size and number [6], our data suggest that the loss of synapses occurs widely throughout the spinal cord and not simply localised to regions where MNs reside. This observation suggests that ALS may impact a broad range of sensory, integratory and motor circuitry. Interestingly, female SOD1^G93a^ mice displayed significantly increased numbers of synapses in dorsal laminae I–II compared to controls, which is supported by previous reports of sensory-level alterations in ALS and other related disorders [22, 76]. p-Ezrin associated tripartite synapse loss also appeared to be most prominent in ventral lamina VII, where the smallest percentage of synapses are contacted by p-Ezrin. It is plausible that greater glial coverage of synapses in some regions of the nervous system may in fact help preserve synapses from degeneration. Regional diversity in astrocyte properties could also contribute to variation in tripartite synapse vulnerability [54, 73]. It would be of interest to investigate the nature of tripartite synapses in regions of the nervous system more resistant to ALS neurodegeneration, such as the oculomotor nucleus [14], to further elucidate why some synapses are more vulnerable than others.

Our mapping analyses also suggested that while synapse and tripartite synapse loss occurred in both male and female SOD1^G93a^ mice, structural changes in PSDs may be more prominent across the whole spinal cord in males. In female SOD1^G93a^ mice however, PSD size was reduced in many ventral laminae but increased in some dorsal laminae. Other studies indicate a more discernible gender bias in SOD1^G93a^ mice, with males showing an earlier onset and increased rate of disease progression, and some changes in specific subtypes of spinal synapses only occurring in males [10, 36, 40]. In addition, clinical ALS cases present with a higher incidence in males [52].

Our extensive analysis of SOD1^G93a^ mouse spinal cords revealed significant postsynaptic changes at the early symptomatic stage of 16 weeks (112 days), with no overt changes at 12 weeks (84 days) or 8 weeks (56 days) of age, although early signs of tripartite synapse loss were observed at 12-weeks. Others have reported reduced sizes of clusters of other synaptic proteins, such as scaffolding proteins Shank1 and Homer, and glutamatergic AMPA receptors on spinal cord MNs in pre-symptomatic 8-week old SOD1^G93a^ mice [3]. Similarly, pre-symptomatic changes in cortical synapses and neuromuscular junctions have been observed as early as 4–6 weeks in mouse models [18, 21, 24, 25, 29]. Our current data does not suggest that significant synaptic alterations occur pre-symptomatically in spinal cord circuits, but instead suggests that they occur at the early symptomatic stages of the disease. Dying-forward (cortex to spinal cord) and dying-backward (periphery to spinal cord) mechanisms exist pre-symptomatically [18, 21], and may not lead to significant synaptic changes in the spinal cord until later in the disease progression. Furthermore, different techniques to visualise and quantify synaptic structure and function may confound direct comparisons across studies. For example, it is conceivable that changes in dendritic spines may occur without any apparent changes in the expression or organisation of PSD95 [25]. Comparative analyses of synaptic changes in both the brain and spinal cord in the early stages of ALS may help decipher whether synaptic changes occur simultaneously in various parts of the CNS, or whether, for example, motor cortex dysfunction drives synaptic dysfunction in the spinal cord in a feedforward manner.

In accordance with other reports of changes in the structure of synapses in ALS [3, 63, 64], our high-resolution imaging revealed reduced PSD size in early symptomatic SOD1^G93a^ males. By employing super-resolution microscopy, we have performed nanoscopic analysis of the molecular organisation of synapses in ALS for the first time. This powerful microscopy technique has characterised the nature of ALS synaptopathy at the sub-diffraction scale, revealing a reduced number of large multi-NC synapses. PSD95 forms nanoscale postsynaptic domains that tether neurotransmitter receptor subdomains at the postsynaptic membrane, which align with presynaptic release sites to create trans-synaptic signalling nanocolumns [70]. Synaptic strength appears to correlate with the number of nanodomains within the synapse [7, 49, 56]. Our previous work has shown that multi-NC synapses are more likely to be tripartite synapses, based on their association with PAP proteins EAAT2 and p-Ezrin [6]. This supports the concept that large multi-NC tripartite synapses are those which are selectively vulnerable to degeneration in ALS. Given that the number of PSD95 subdomains per synapse correlates with synaptic strength, it is possible that the loss of these large, high-fidelity synaptic connections significantly contributes to early stage, progressive motor deficits. zDHHC2 is a palmitoylating enzyme that facilitates the clustering of PSD95 at the synapse. From our data, we also speculate that the dissociation of zDHHC2 from the PSD may lead to reduced clustering of PSD95 at the synapse [30], though the precise role of protein palmitoylation in neurodegenerative disease remains to be determined [12, 84].

Importantly, we were able to replicate our key observations of synapse and tripartite synapse loss in ALS mouse models when studying human cervical spinal cord tissue from ALS patients. Our analysis suggested that C9orf72 patients displayed significant synaptic loss and tripartite synapse vulnerability, while SOD1 patients did not show significant synapse or tripartite synapse loss compared to controls. In comparison, a previous investigation into synaptic changes in the prefrontal cortex of ALS patients revealed no differences in synaptic loss between SOD1 and C9orf72 associated cases, but did suggest that synaptic loss was most significant in cases displaying TDP-43 pathology—of which SOD1 cases showed none [38]. Together these data highlight the heterogenous nature of ALS. This heterogeneity is further underlined by the range of familial and sporadic cases of ALS. While less data is currently available regarding the fate of synapses in sporadic ALS, given that C9orf72 mutations represent the most common genetic basis of both sporadic and familial ALS and since three out of five C9orf72 patients used in this study had no family history of ALS, our findings may be applicable to both familial and sporadic cases.

On the face of it, our data presents a dichotomy between the SOD1 and C9orf72 mouse models and human patients. However, the lack of a robust phenotype in the C9orf72 mouse model (in our hands) likely renders it inappropriate to directly compare the two genotypes across mouse models and human patients. In addition, it is already known that there are differences between mouse lines expressing ALS-linked mutations and the human genetic cases that these models are attempting to recapitulate. For example, the SOD1^G93a^ model shows a rapid rate of disease onset compared to the slow progression observed in human SOD1^G93a^ cases [61]. Furthermore, the reported presence of TDP-43 pathology in SOD1^G93a^ mice at end stage conflicts with the lack of such pathology in human SOD1 cases [50, 67]. Together with our own data, this demonstrates that caution should be taken when attempting to directly correlate mouse models with human cases involving similar genetic origins. It should be noted that while the SOD1^G93a^ mouse may not fully recapitulate human ALS, it nevertheless represents a useful entry point for pursuing mechanistic understanding of the disease. Indeed the use of such mouse models has been essential in many advances in the understanding of ALS pathology [34].

The mechanisms leading to tripartite synapse degeneration remain to be determined. Mislocalisation and aggregation of TDP-43 in the cytoplasm of both neurons and glia is correlated with synapse loss in human ALS [38], and could play a role in designating tripartite synapses for degeneration. However, there is mixed evidence of TDP-43 pathology in the SOD1^G93a^ mouse model [43, 67, 69]. Alternatively, the loss of astrocytic EAAT2 in ALS, due to aberrant RNA processing, is known to induce glutamatergic excitotoxicity that could lead to synapse degeneration and ultimately cell death [41, 47, 51, 62]. Our own data supports this hypothesis given that synaptic loss coincides with EAAT2 loss. Astrocytes also contribute to bi-directional signalling mechanisms that help modulate spinal cord mediated motor behaviour through purinergic signalling [1, 8, 9, 11, 79, 80]. Neuronal-derived glutamate released during locomotor network activity activates astrocytes through mGluR5 receptors, leading to ATP/adenosine release from astrocytes that inhibits neuronal activity through A_1_ adenosine receptors [8]. Increased adenosine levels in ALS patients [83], combined with increased expression of the excitatory A_2A_ adenosine receptors [57], could imbalance this modulatory mechanism, biasing the system toward excitotoxic actions at the tripartite synapse. Our current study provides evidence that tripartite synapses, and the specific signalling pathways they utilise to regulate neuronal activity, may act as a fulcrum of ALS. These novel findings regarding selective synaptic vulnerability highlight numerous future avenues to explore and provide the framework to investigate the mechanisms and therapeutic potential of ALS tripartite synaptopathy.

## Supplementary Information

Below is the link to the electronic supplementary material.

Supplementary file1 (PDF 1352 KB)

## Data Availability

The data used in this manuscript is freely available upon request.
