# Supplementary material for: Selective vulnerability of tripartite synapses in amyotrophic lateral sclerosis
Source: Acta Neuropathol. 2022 Mar 19;143(4):471–86. doi: 10.1007/s00401-022-02412-9 (PMC8960590; doi:10.1007/s00401-022-02412-9)
Supplement: Supplementary file 1 — Supplementary file1 (PDF 1352 KB) [file 401_2022_2412_MOESM1_ESM.pdf]

# Supplementary Figures And Tables

**Selective Vulnerability of Tripartite Synapses in Amyotrophic Lateral Sclerosis.**

Broadhead MJ[*1,2], Bonthron C*[1], Waddington J[1], Smith WV[1], Lopez MF[1], Burley S[1], Valli J[2], Zhu F[3], Komiyama NH[3,4], Smith C[5,6] Grant SGN[3,4], Miles GB[†1].

* co-first author

1. School of Psychology and Neuroscience, University of St Andrews, St Andrews, UK
2. Edinburgh Super-Resolution Imaging Consortium, Heriot Watt University, Edinburgh, UK.
3. Genes to Cognition Program, Centre for Clinical Brain Sciences, University of Edinburgh, Edinburgh EH16 4SB, UK.
4. Simons Initiative for the Developing Brain (SIDB), Centre for Discovery Brain Sciences, University of Edinburgh, Edinburgh EH8 9XD, UK.
5. Centre for Clinical Brain Sciences, University of Edinburgh, Edinburgh, UK
6. Euan MacDonald Centre for MND Research, The University of Edinburgh, Edinburgh, UK.

Revised Version: 2022/02/19

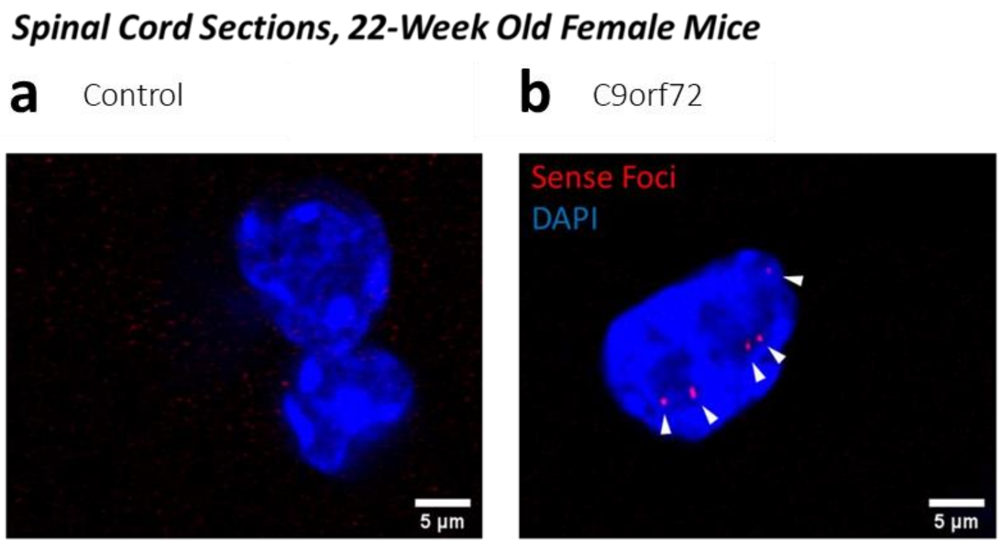


**SI. Figure 1**. **G4C2-repeat RNA foci in C9orf72 mouse spinal cord tissue**. Foci are labelled by FISH using Cy5-conjugated RNA probes (red) and appear visible in DAPI-labelled cell nuclei (blue). **a-b**. RNA foci are present in the nuclei of cells in spinal cord sections from a 22-week old female PSD95-eGFP mouse expressing the C9orf72 mutation, but not in an age and gender matched control mouse.

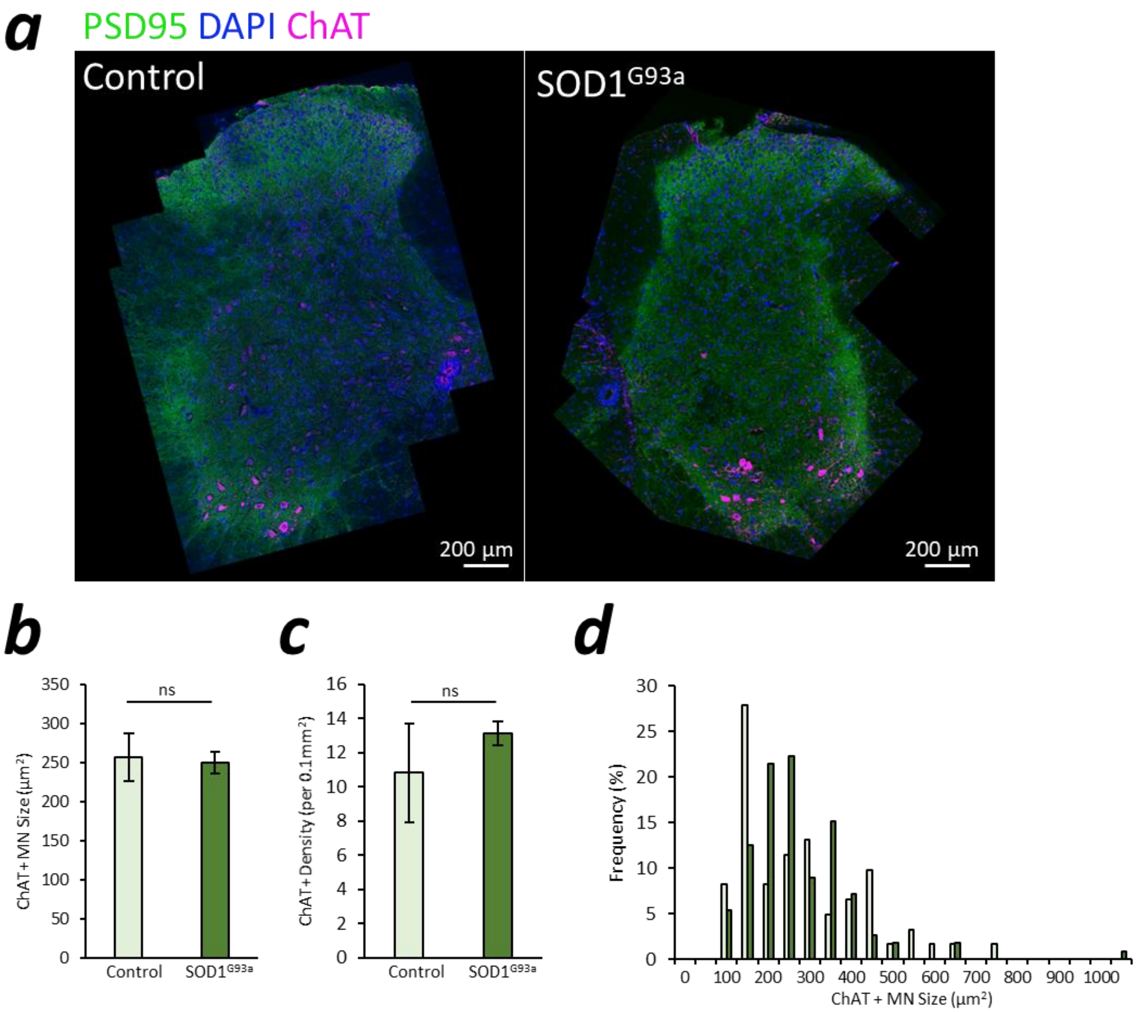


**SI. Figure 2. ChAT immunolabelling of 16 week SOD1$^{G93a}$ mice reveals no significant loss of MNs**. **a**. Example high-resolution maps of PSD95-eGFP, ChAT and DAPI labelling in control and SOD1$^{G93a}$ 16 week male spinal cords. **b**. Chart plotting MN size (area) in Control and SOD1$^{G93a}$ mice. **c**. Chart plotting MN cell density in Control and SOD1$^{G93a}$ mice. **d**. Frequency histogram plotting the number of cells of a given size in Control and SOD1$^{G93a}$ mice.

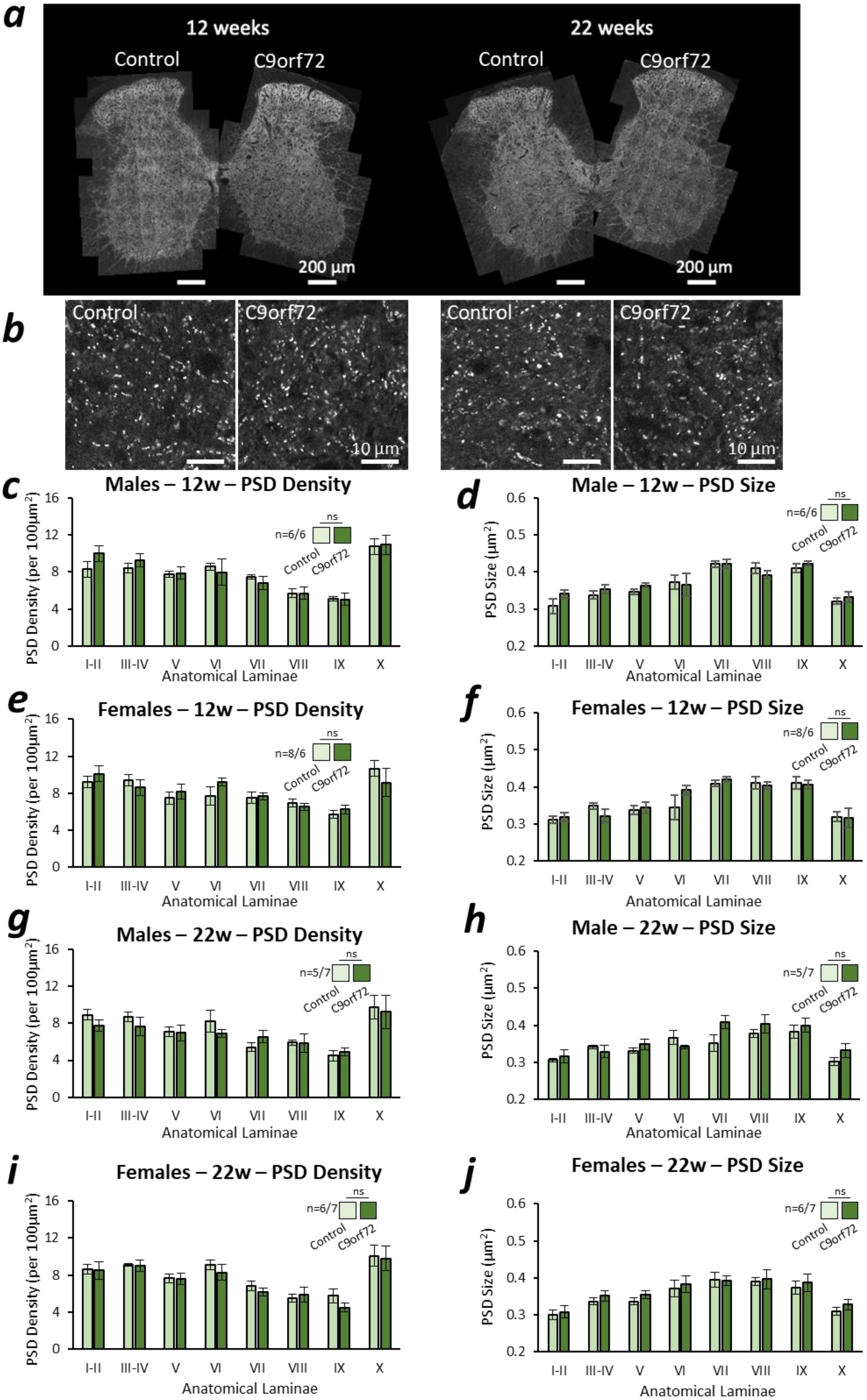

a
12 weeks
22 weeks
Control
C9orf72
200 µm
b
10 µm
c
Males – 12w – PSD Density
d
Male – 12w – PSD Size
e
Females – 12w – PSD Density
f
Females – 12w – PSD Size
g
Males – 22w – PSD Density
h
Male – 22w – PSD Size
i
Females – 22w – PSD Density
j
Females – 22w – PSD Size
PSD Density (per 100µm²)
PSD Size (µm²)
Anatomical Laminae
I-II
III-IV
V
VI
VII
VIII
IX
X
ns
n=6/6
n=8/6
n=5/7
n=6/7

**SI. Figure 3. Widescale mapping of excitatory synapses reveals no changes associated with C9orf72 mutation in C9BAC500 mice. a.** High-resolution maps of PSD95-eGFP expression in 12 week and 22 week old C9orf72 mice, and controls. **b**. Example cropped high resolution images of individual PSDs in C9orf72 mice and controls. **c**. Chart plotting the PSD density in male 12 week old mice. **d**. Chart plotting the PSD size in male 12 week old mice. **e**. Chart plotting the PSD density in female 12 week old mice. **f**. Chart plotting the PSD size in female 12 week old mice. **G**. Chart plotting the PSD density in male 22 week old mice. **h**. Chart plotting the PSD size in male 22 week old mice. **i.** Chart plotting the PSD density in female 22 week old mice. **j.** Chart plotting the PSD size in female 22 week old mice.

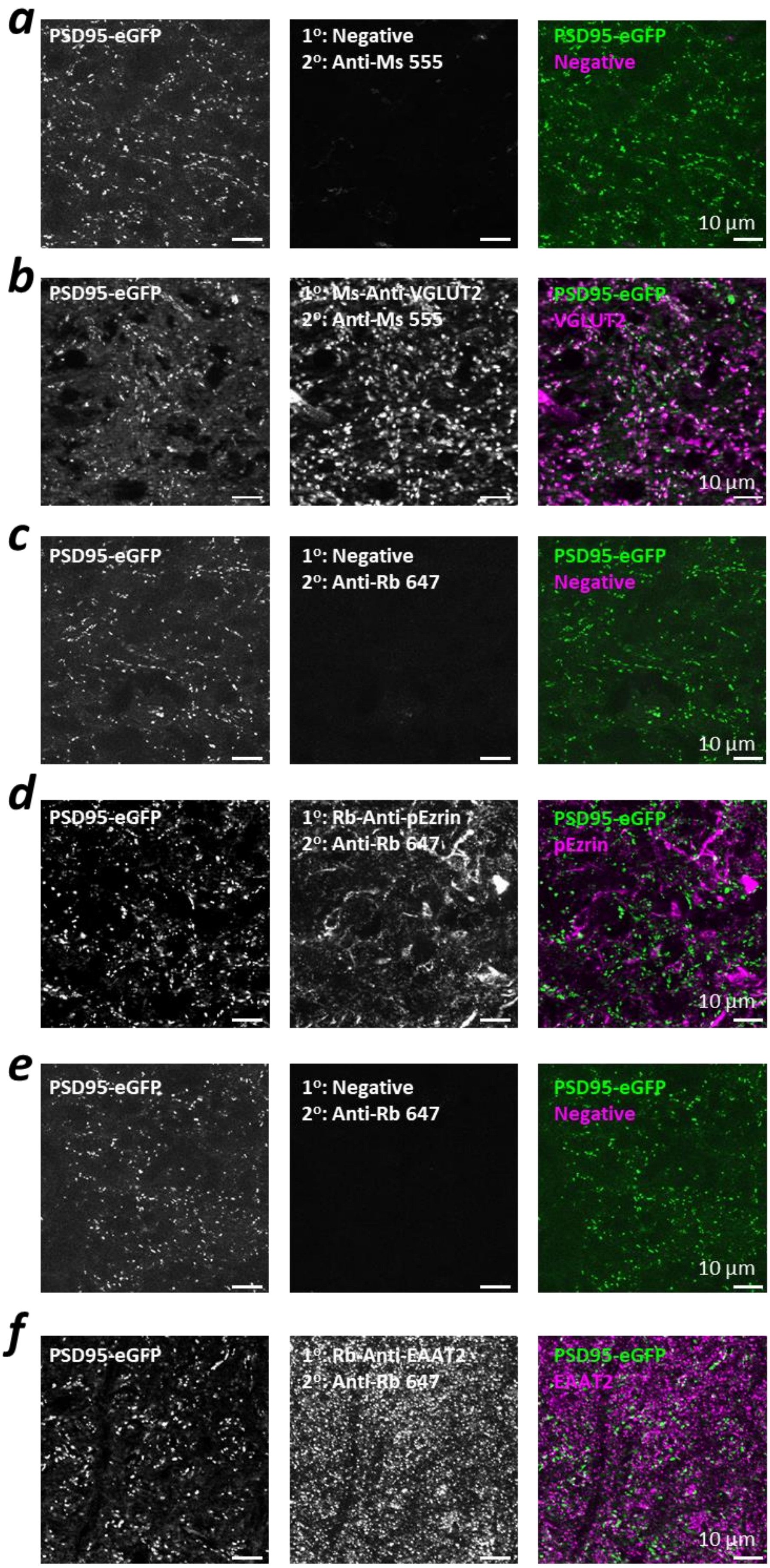
a
PSD95-eGFP
1º: Negative
2º: Anti-Ms 555
PSD95-eGFP
Negative
10 µm
b
PSD95-eGFP
1º: Ms-Anti-VGLUT2
2º: Anti-Ms 555
PSD95-eGFP
VGLUT2
10 µm
c
PSD95-eGFP
1º: Negative
2º: Anti-Rb 647
PSD95-eGFP
Negative
10 µm
d
PSD95-eGFP
1º: Rb-Anti-pEzrin
2º: Anti-Rb 647
PSD95-eGFP
pEzrin
10 µm
e
PSD95-eGFP
1º: Negative
2º: Anti-Rb 647
PSD95-eGFP
Negative
10 µm
f
PSD95-eGFP
1º: Rb-Anti-EAAT2
2º: Anti-Rb 647
PSD95-eGFP
EAAT2
10 µm

**SI. Figure 4. Mouse immunohistochemistry staining controls.**

Negative primary (1°) antibody staining controls were performed in PSD95-eGFP mouse spinal cord sections, with secondary (2°) antibodies included to assess whether the resultant immunofluorescence signal was due to background or non-specific 2° labelling. **a**. Negative primary control for VGLUT2, displaying reference PSD95-eGFP image, negative 1° control and merged image (left-right). **b**. Positive primary for VGLUT2, displaying reference PSD95-eGFP image, positive 1° control and merged image. **c**. Negative primary control for p-Ezrin, displaying reference PSD95-eGFP image, negative 1° control and merged image. **d**. Positive primary for p-Ezrin, displaying reference PSD95-eGFP image, positive 1° control and merged image. **e**. Negative primary control for EAAT2, displaying reference PSD95-eGFP image, negative 1° control and merged image. **f**. Positive primary for EAAT2, displaying reference PSD95-eGFP image, positive 1° control and merged image.

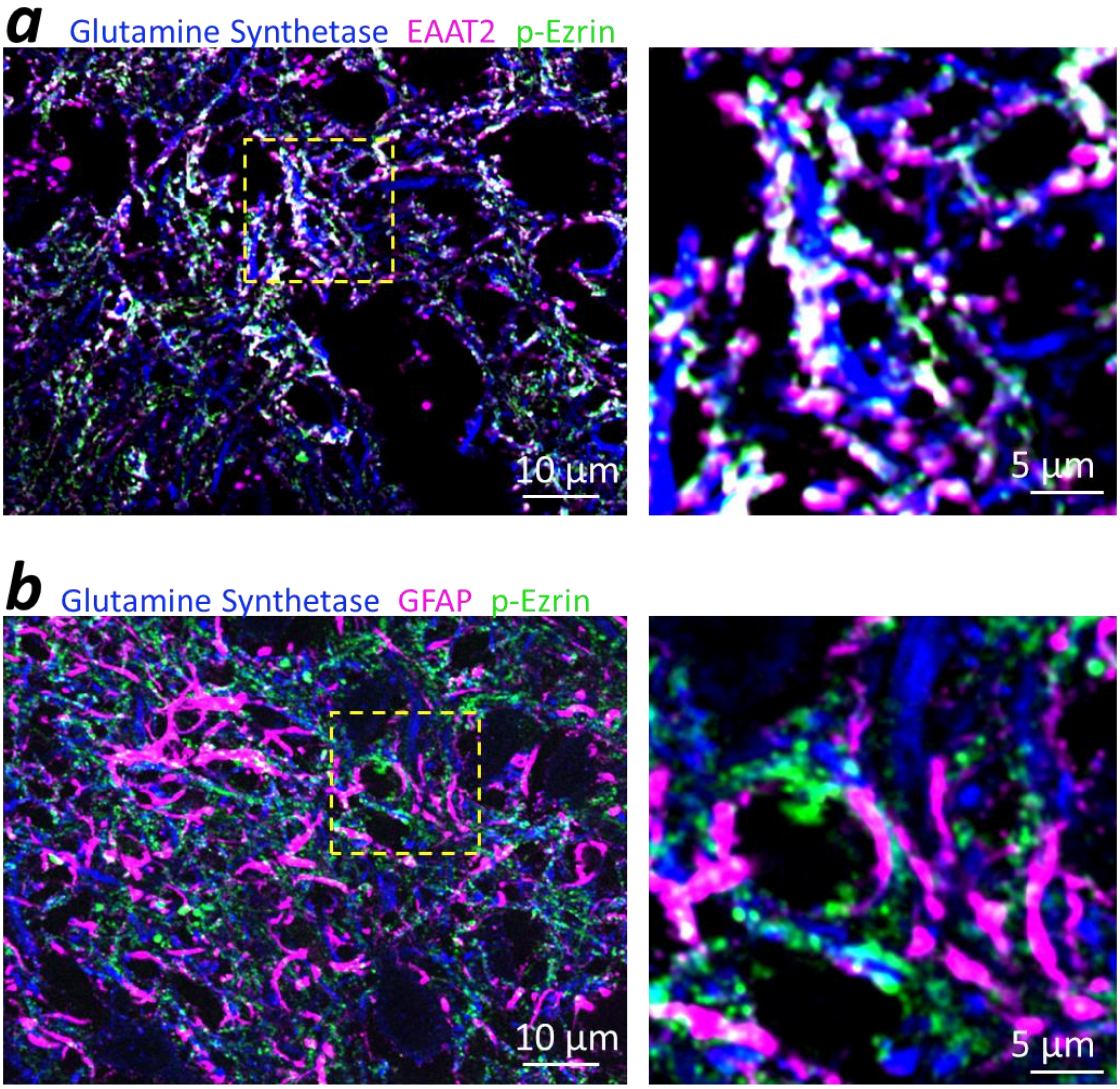


**SI. Figure 5. P-Ezrin and EAAT2 label perisynaptic astrocytic processes in the mouse spinal cord.** Spinal cord sections from a P90 C57Bl/6J mouse were immunolabelled for astrocytic markers using a combination of conventional primary-secondary antibody labelling and dye-conjugated primary antibody labelling. Glutamine synthetase was visualised with a FITC-conjugated secondary antibody, GFAP and EAAT2 were conjugated directly to Alexa Fluor 594, and p-Ezrin was conjugated directly to Alexa Fluor 647. **a**. p-Ezrin and EAAT closely colocalise along glutamine synthetase-positive astrocyte branches. **b**. p-Ezrin puncta typically associate along glutamine synthetase branches as opposed to GFAP-positive branches, which are more associated with larger astrocytic branches and the soma.

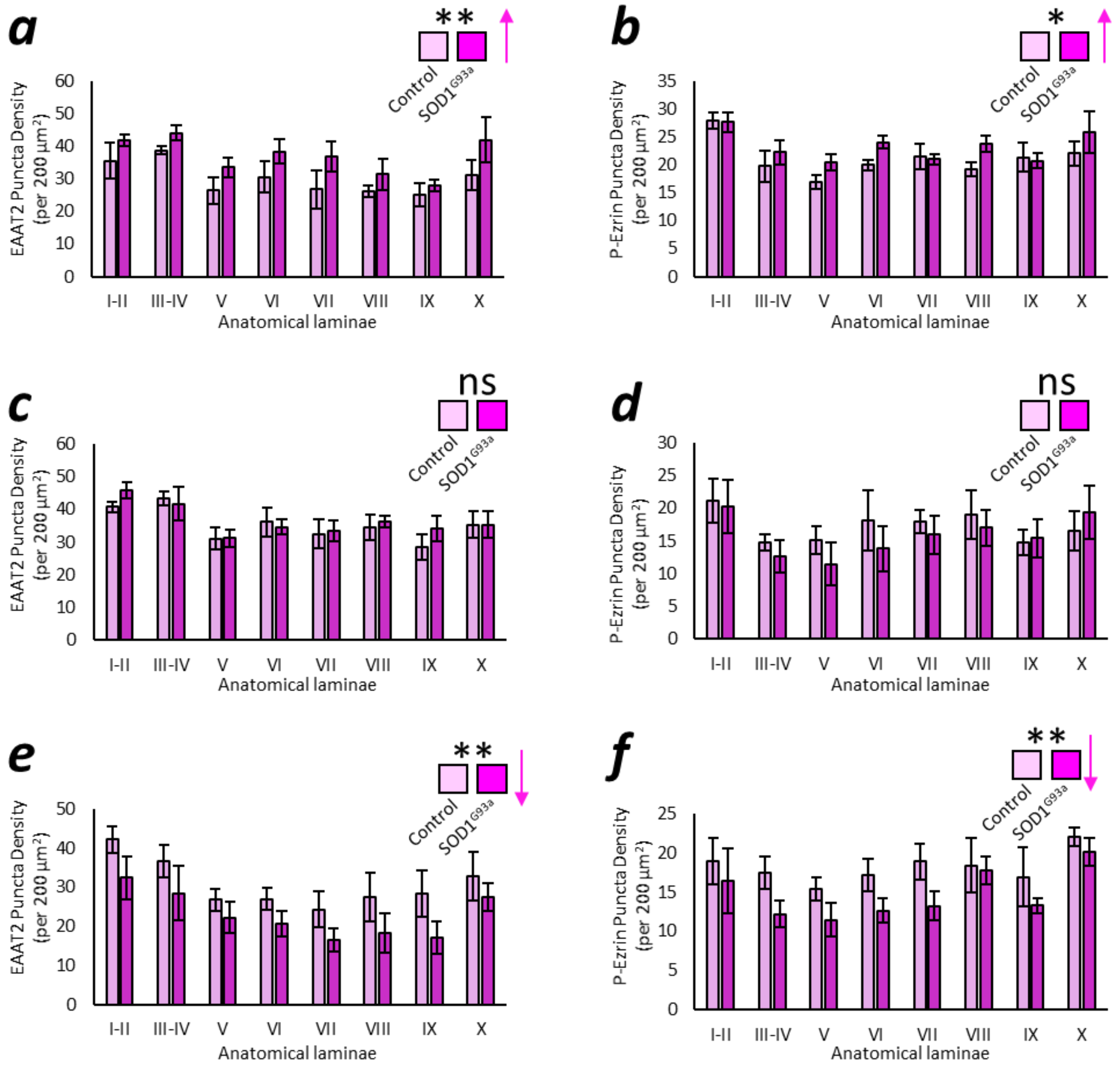


**SI. Figure 6. Mapping PAP marker distribution in the ALS mouse spinal cord reveals age-dependent changes in ALS.** The puncta density of EAAT2 (**a, c, e**) and p-Ezrin (**b, d, f**) were analysed for each spinal cord laminae at 8 weeks (**a-b**), 12 weeks (**c-d**) and 16 weeks (**e-f**). These data reveal increased PAP protein expression at the pre-symptomatic 8 week stage but significantly reduced expression at the early symptomatic 16 week stage.

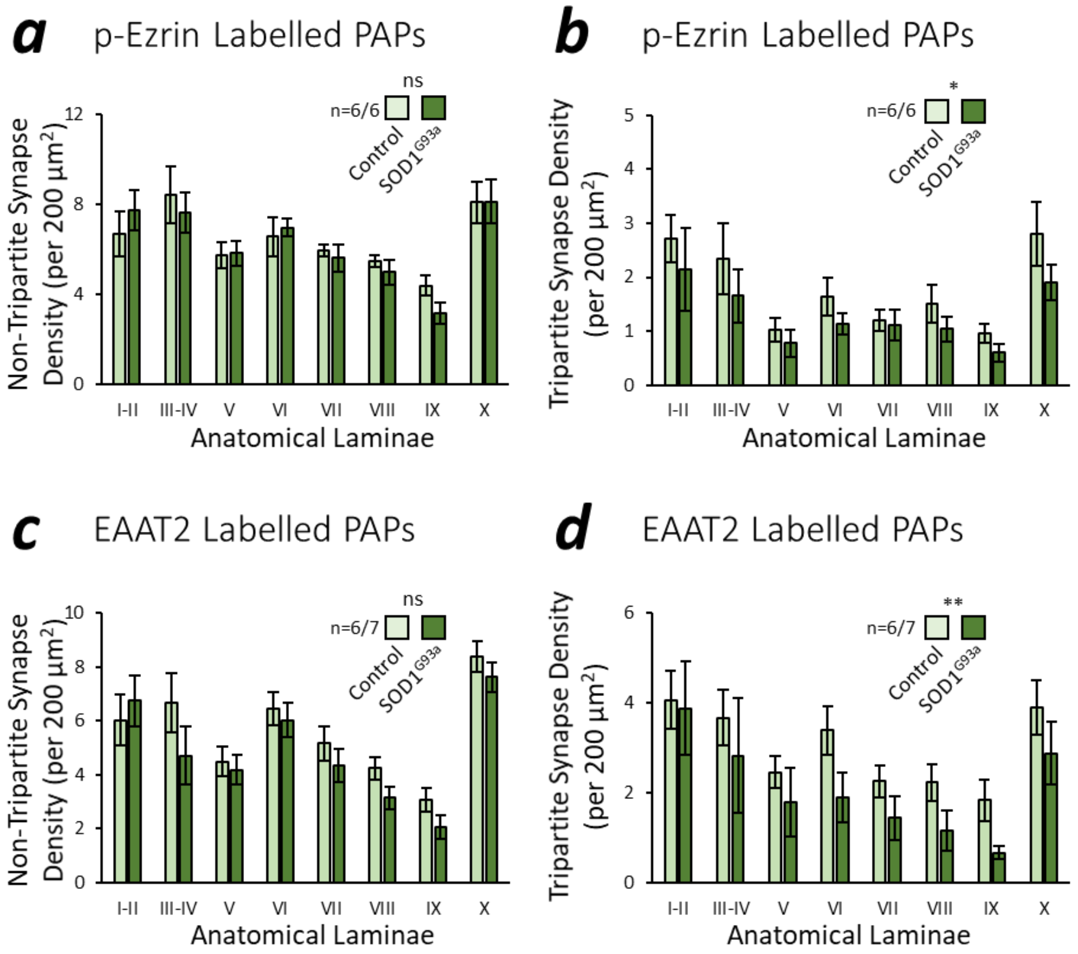


**SI. Figure 7. Quantifying Tripartite Synapses in the 16 Week Old Female SOD1$^{G93a}$ Mice.** Spinal cord sections from 16 week old female SOD1$^{G93a}$ mice and controls were immunolabelled for VGLUT2 and either p-Ezrin (**a-b**) or EAAT2 (**c-d**) to quantify tripartite synapses using high-resolution anatomical mapping. There were no differences between controls and SOD1$^{G93a}$ mice in the density of non-tripartite synapses across the spinal cord maps (**a, c**). p-Ezrin associated synapses (**b**) and EAAT2-associated synapses (**d**) were significantly reduced in their number in SOD1$^{G93a}$ mice compared to controls

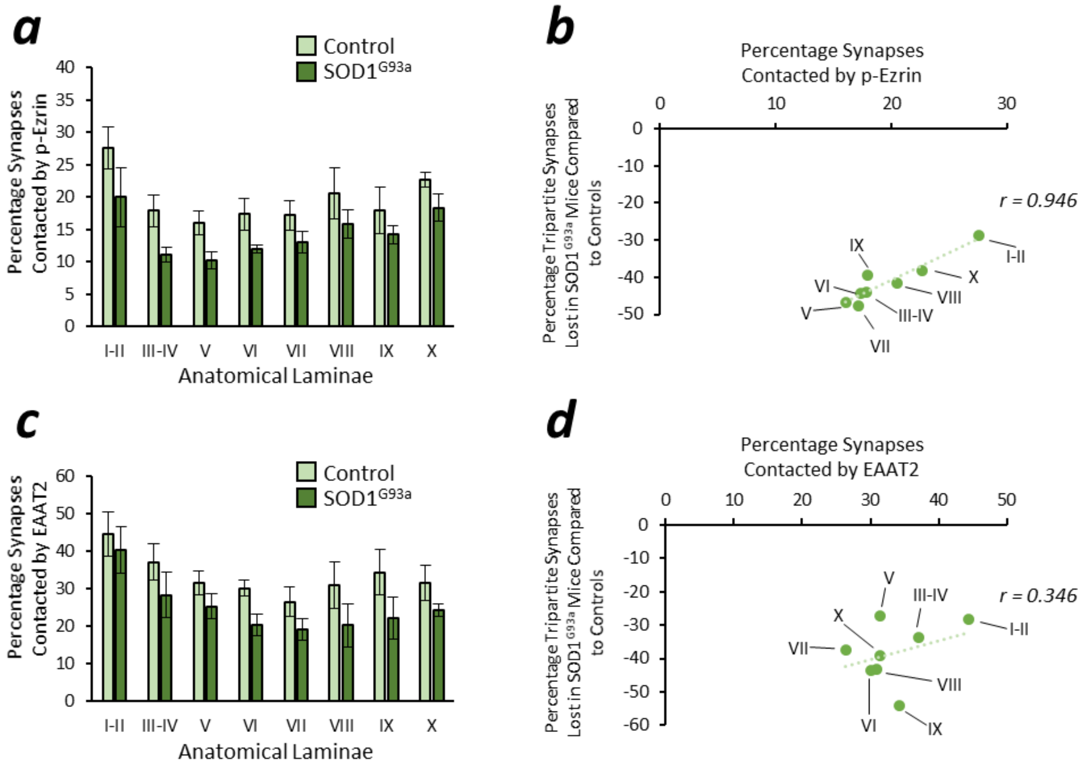


**SI. Figure 8. Regional tripartite synaptic loss correlates astrocytic contact with synapses**. **a.** The percentages of synapses (PSD95-eGFP and VGLUT2 presynaptic terminals) contacted by p-Ezrin in controls and SOD1$^{G93a}$ 16 week old males in each lamina, showing diversity in the percentage of tripartite synapses between different anatomical laminae. **b.** The percentage loss of p-Ezrin tripartite synapses for each lamina, calculated from the density of tripartite synapses in SOD1$^{G93a}$ and control mice, is plotted against the percentage of synapses contacted by p-Ezrin in each lamina. **c.** The percentages of synapses contacted by EAAT2 in controls and SOD1$^{G93a}$ 16 week old males in each lamina, showing diversity in the percentage of tripartite synapses between different anatomical laminae. **d.** The percentage loss of EAAT2 tripartite synapses for each lamina, calculated from the density of tripartite synapses in SOD1$^{G93a}$ and control mice, is plotted against the percentage of synapses contacted by EAAT2 in each lamina.

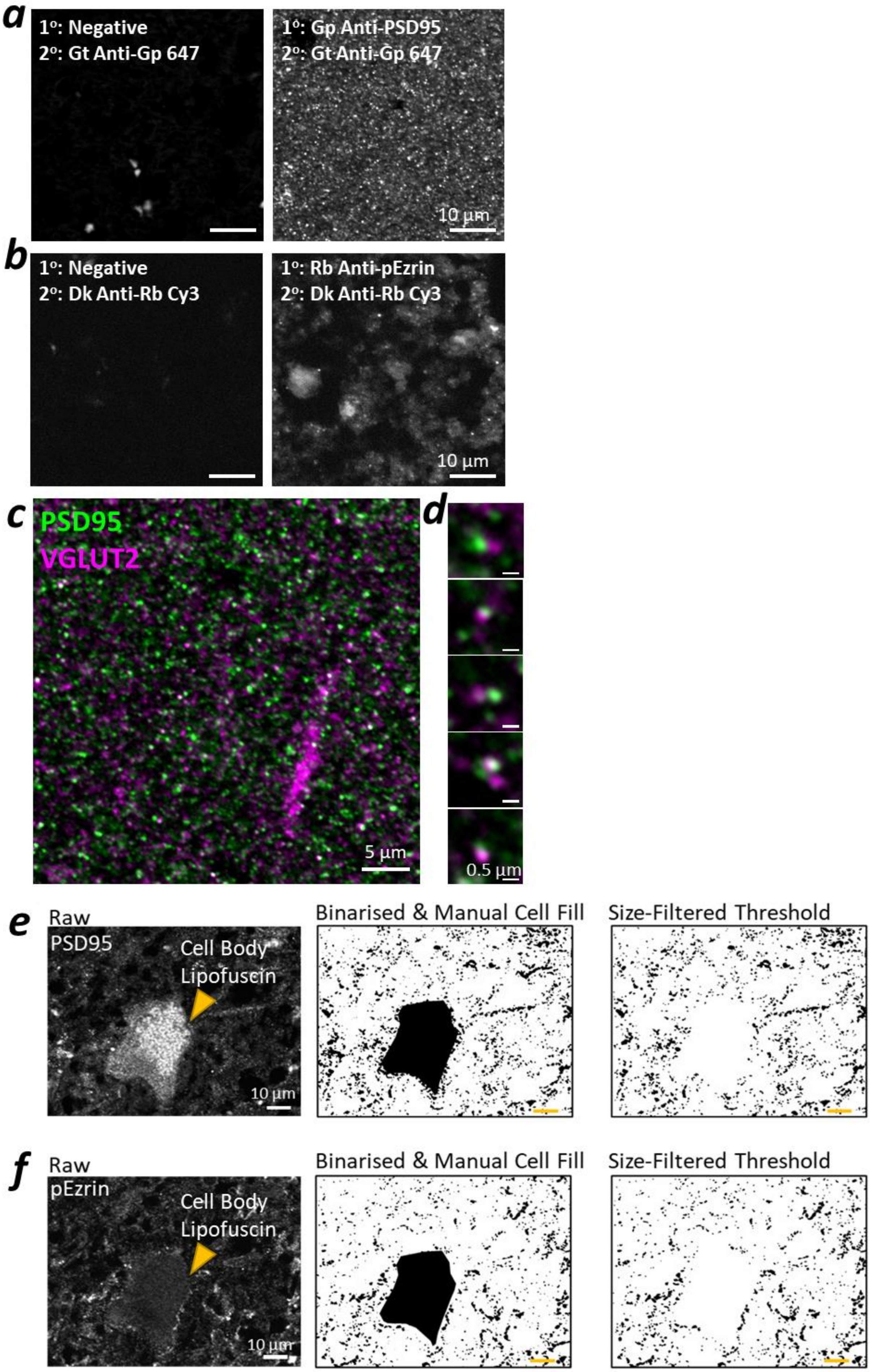
a
1°: Negative
2°: Gt Anti-Gp 647
1°: Gp Anti-PSD95
2°: Gt Anti-Gp 647
10 µm
b
1°: Negative
2°: Dk Anti-Rb Cy3
1°: Rb Anti-pEzrin
2°: Dk Anti-Rb Cy3
10 µm
c
PSD95
VGLUT2
5 µm
d
0.5 µm
e
Raw
PSD95
Cell Body
Lipofuscin
10 µm
Binarised & Manual Cell Fill
Size-Filtered Threshold
f
Raw
pEzrin
Cell Body
Lipofuscin
10 µm
Binarised & Manual Cell Fill
Size-Filtered Threshold

**SI. Figure 9. Human Immunohistochemistry Staining Controls.**

Human hippocampal post-mortem sections were used to test antibody labelling of PSD95 and p-Ezrin. **a-b.** Left panel shows negative-primary (1$^o$) labelling along with fluorescent secondary (2$^o$) antibody labelling. Right hand panels show 1$^o$ and 2$^o$ immunolabelling. Images displayed at the same background/contrast adjustment. The fluorescent 2$^o$ antibodies therefore result in little background labelling without the respective 1$^o$ antibodies. **c.** Co-labelling VGLUT2 and PSD95 in human hippocampal tissue reveals juxtaposed pre and postsynaptic puncta, indicative of bona-fide synapses (examples shown in panel **d**). **e-f.** Panels demonstrating the semi-automated analysis of **e**) synapses (PSD95 labelling) and **f**) PAPs (p-Ezrin labelling). Raw images undergo image processing and are manually thresholded by the user to create binarized images. Large cell bodies with high levels of autofluorescence (orange arrowhead), when present in the image, are manually delineated and filled. The final structures analysed undergo a size filter (8-500 pixels total for PSD95, 8-2000 pixels total for p-Ezrin). This final size filter removes low levels of background noise, large cell bodies and other large structures that are unlikely to be true synapses or PAPs.

# Supplementary Tables

| SI. Table 1. Human Post-Mortem Tissue MRC ID's | | | | | |
|---|---|---|---|---|---|
| **MRC Brain Bank ID** | **Sex** | **Age** | **Genotype/Condition** | **Tissue Type** | **Purpose** |
| BBN001.29085 | Male | 46 | Control | Cervical Spinal Cord | Tripartite Synapse Labelling (Fig. 4) |
| BBN001.29084 | Male | 58 | Control | Cervical Spinal Cord | Tripartite Synapse Labelling (Fig. 4) |
| BBN001.26797 | Male | 49 | Control | Cervical Spinal Cord | Tripartite Synapse Labelling (Fig. 4) |
| BBN001.26309 | Male | 69 | Control | Cervical Spinal Cord & Hippocampus | Tripartite Synapse Labelling (Fig. 4) & IHC Protocol Validation (SI. Fig 9) |
| BBN001.25751 | Male | 50 | Control | Cervical Spinal Cord | Tripartite Synapse Labelling (Fig. 4) |
| BBN001.29824 | Female | 71 | Control | Cervical Spinal Cord | Tripartite Synapse Labelling (Fig. 4) & IHC Protocol Validation (SI. Fig 9) |
| BBN_20613 | Male | 50 | C9orf72 (ALS) | Cervical Spinal Cord | Tripartite Synapse Labelling (Fig. 4) |
| BBN_20993 | Male | 43 | C9orf72 (ALS) | Cervical Spinal Cord | Tripartite Synapse Labelling (Fig. 4) |
| BBN001.28792 | Male | 58 | C9orf72 (ALS) | Cervical Spinal Cord | Tripartite Synapse Labelling (Fig. 4) |
| BBN001.35827 | Male | 66 | C9orf72 (ALS) | Cervical Spinal Cord | Tripartite Synapse Labelling (Fig. 4) |
| BBN001.35506 | Male | 71 | C9orf72 (ALS) | Cervical Spinal Cord | Tripartite Synapse Labelling (Fig. 4) |
| BBN001.36134 | Male | 45 | SOD1 (ALS) | Cervical Spinal Cord | Tripartite Synapse Labelling (Fig. 4) |
| BBN001.35135 | Male | 71 | SOD1 (ALS) | Cervical Spinal Cord | Tripartite Synapse Labelling (Fig. 4) |
| BBN001.34243 | Male | 53 | SOD1 (ALS) | Cervical Spinal Cord | Tripartite Synapse Labelling (Fig. 4) |
| BBN001.29543 | Male | 47 | SOD1 (ALS) | Cervical Spinal Cord | Tripartite Synapse Labelling (Fig. 4) |
| BBN00135209 | Female | 70 | SOD1 (ALS) | Lumbar Spinal Cord | IHC Protocol Validation (data not shown) |
| BBN001.29825 | Female | 70 | C9orf72 (ALS) | Lumbar Spinal Cord | IHC Protocol Validation (data not shown) |
